# Oleanolic acid-derived triterpenoids with dual TGR5 agonistic and RORγ inverse agonistic activity

**DOI:** 10.1039/d6md00471g

**Published:** 2026-10-06

**Authors:** Panagiota Stamou, Alexander F. Perhal, Patrik F. Schwarz, Jorge Enrique Hernández González, Justin Weal, Ya Chen, Alexios Leandros Skaltsounis, Maria Halabalaki, Johannes Kirchmair, Verena M. Dirsch, Ioannis K. Kostakis

**Affiliations:** a Department of Pharmacy, Division of Pharmaceutical Chemistry, National and Kapodistrian University of Athens Panepistimiopolis, Zografou 15771 Athens Greece ikkostakis@pharm.uoa.gr; b Department of Pharmaceutical Sciences, University of Vienna 1090 Vienna Austria; c Multiuser Center for Biomolecular Innovation, São Paulo State University – UNESP São José do Rio Preto SP Brazil; d Department of Pharmacy, Division of Pharmacognosy and Natural Products Chemistry, National and Kapodistrian University of Athens Panepistimiopolis Zografou Athens 15771 Greece

## Abstract

The Takeda G protein-coupled receptor 5 (TGR5) and the retinoid-related orphan receptor γ (RORγ) are key regulators of metabolic and immune pathways. This makes them attractive targets for developing anti-inflammatory and metabolic therapeutics. In this study, we designed and synthesised a series of oleanolic acid-derived triterpenoid derivatives to explore their potential as dual modulators of these receptors. The focus of the structural modifications was on the double bond, C-3 substitution, and A-ring rearrangements in order to investigate their impact on biological activity and establish structure–activity relationships within the pentacyclic triterpene scaffold. Biological evaluation using CRE–luciferase reporter assays identified oleanolic acid (1), oleanonic acid (5), and compounds 7 and 8 as TGR5 agonists with concentration-dependent activities. Oleanolic acid (1) and the newly identified seco-derivative 8 displayed the most favourable potency and efficacy profiles. Gal4–RORγ reporter assays demonstrated inverse agonistic activity for oleanolic acid (1) and compounds 7 and 8. Although 7 and 8 exhibited lower potency than 1, both retained pronounced inhibitory efficacy, indicating that substantial scaffold rearrangement remains compatible with RORγ modulation. A resazurin conversion assay showed no evidence of cytotoxicity under the conditions tested, supporting that the observed receptor effects were not attributable to general cytotoxicity. Molecular modelling and Nwat-MMGBSA calculations supported the experimental findings and emphasized the role of conserved buried water molecules in stabilising ligand–receptor interactions in both targets. Overall, these findings identify oleanolic acid-derived triterpenoids as promising scaffolds for dual TGR5/RORγ modulation and provide valuable insights for the future development of multitarget-directed ligands targeting inflammatory and metabolic disorders.

## Abstract 2

Oleanolic acid-derived seco-triterpenoid 8 retains dual TGR5 agonistic and RORγ inverse agonistic activity despite extensive A-ring rearrangement.

## Graphical abstract

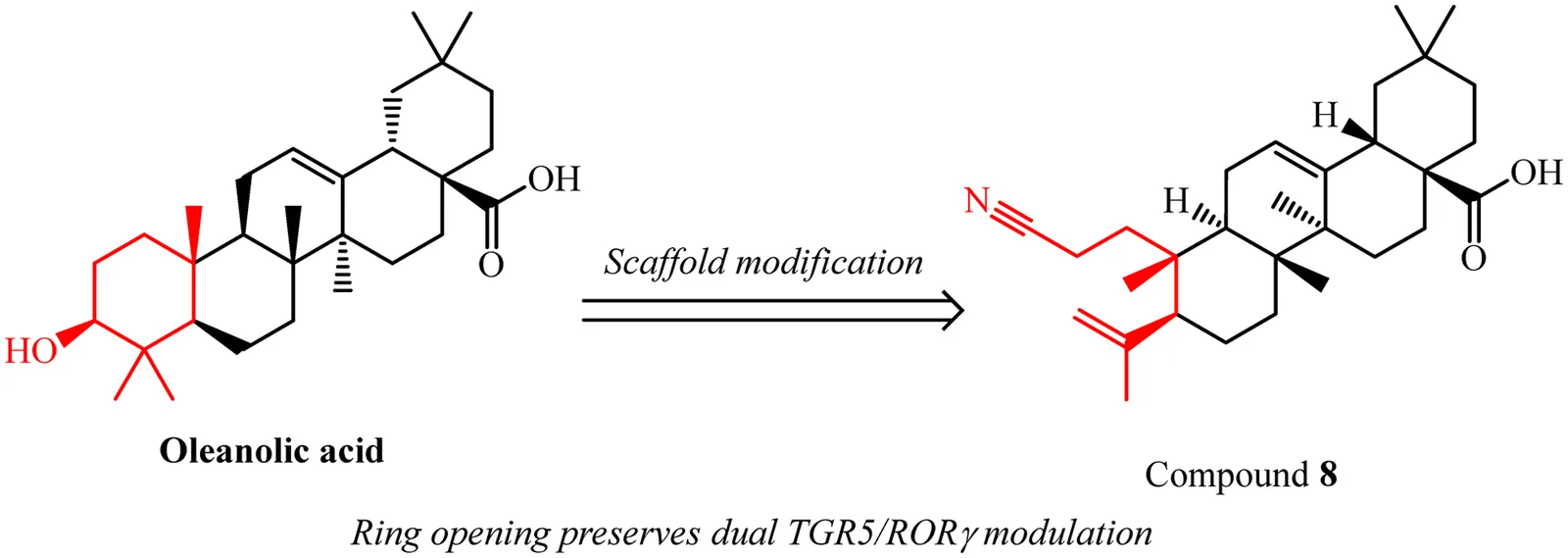

## 1. Introduction

Takeda G protein-coupled receptor 5 (TGR5), also known as GPBAR-1, belongs to the family of Gα_s_-coupled G protein-coupled receptors (GPCRs). Upon receptor activation, intracellular signalling cascades are initiated, leading to increased cyclic adenosine monophosphate (cAMP) levels and subsequent activation of protein kinase A (PKA).^1,2^ PKA phosphorylates the transcription factor cAMP response element-binding protein (CREB), enhancing the expression of CRE-regulated genes associated with anti-inflammatory activities in immune cells.^3^ These activities include upregulation of the anti-inflammatory cytokine interleukin-10 (IL-10) and suppression of pro-inflammatory mediators such as IL-1β, IL-6, and inducible nitric oxide synthase (iNOS).^4,5^ Additionally, increased cAMP levels diminish the activity of IκB kinases (IKKs), which are normally activated by pro-inflammatory stimuli like TNF-α or lipopolysaccharide (LPS). This suppression reduces IκB phosphorylation, thereby preventing IκB degradation and retaining the nuclear factor κB (NFκB) complex in the cytosol in a transcriptionally inactive state.^6^

Beyond its role in immune regulation, TGR5 activation contributes to metabolic control. By promoting the release of glucagon-like peptide-1 (GLP-1), TGR5 regulates blood glucose levels and supports glycemic balance.^7–9^ TGR5 has thus emerged as a promising therapeutic target for both metabolic disorders and inflammatory diseases, owing to its high expression in immune cells.^3,10^

Bile acids are the endogenous ligands of TGR5. Microbiome-derived secondary bile acids, such as deoxycholic acid (DCA) and lithocholic acid (LCA), have a higher receptor affinity than primary bile acids, including cholic acid (CA) and chenodeoxycholic acid (CDCA).^11^ Interestingly, certain oxo-derivatives of bile acids, such as 3-oxo-LCA, interact directly with Retinoid-related orphan receptor γ (RORγt), a nuclear receptor encoded by the RORC gene on chromosome 1q21.3.^12^ RORγ exists in two isoforms: RORγ1, which regulates circadian rhythms and metabolic gene expression in adipose tissue and liver, and RORγt, expressed in subsets of lymphoid-derived immune cells, including T helper 17 (Th17) cells.^13–17^ While TGR5 is mainly expressed in myeloid cells, RORγt plays a central role in adaptive immunity by driving the production of pro-inflammatory cytokines.^18^ Oxo-bile acid derivatives act as inverse agonists of RORγt, suppressing its transcriptional activity and reducing pro-inflammatory gene expression such as IL-17A, thereby modulating immune responses.^19^

The interplay between these two receptor signalling pathways has prompted us to identify and further optimize natural products as promising modulators of both pathways^20^ several steroidal natural products have been identified as strong TGR5 agonists, including pentacyclic triterpenes such as oleanolic acid, betulinic acid, and ursolic acid.^21–23^ Additionally, also non-steroidal agonists and positive allosteric modulators (PAMs) have recently been reported for TGR5.^4,24^ Interestingly, oleanolic acid and related pentacyclic triterpenes also act as inverse agonists of RORγ,^25–29^ suppressing RORγt-mediated transcription of IL17A and IL17F *in vitro*,^30^ which contributes to their anti-inflammatory and anti-asthmatic effects.^31–33^ Based on these observations, the present study investigates the impact of systematic structural modification of the oleanolic acid scaffold on dual TGR5 and RORγ modulation, aiming to define structure–activity relationships and to identify triterpenoid derivatives with balanced multitarget activity. Specifically, three types of structural variation were targeted (Fig. 1): (i) isomerization of the Δ12 double bond to the more thermodynamically stable Δ13(18) position, to investigate the impact of this conformational change on the ability of the pentacyclic scaffold to engage both receptors, (ii) oxidation of the C-3 hydroxyl to the corresponding ketone and subsequent C-28 amide formation, to assess the contribution of C-3/C-28 polarity and hydrogen-bonding capacity to activity, and (iii) A-ring oxime formation followed by Beckmann rearrangement to produce either a ring-opened nitrile or a ring-expanded lactam. This introduces new polar functionality and an altered A-ring conformation at a position that, based on the co-crystallized ligands of both receptors, lies close to the pocket entrance.

**Fig. 1:**
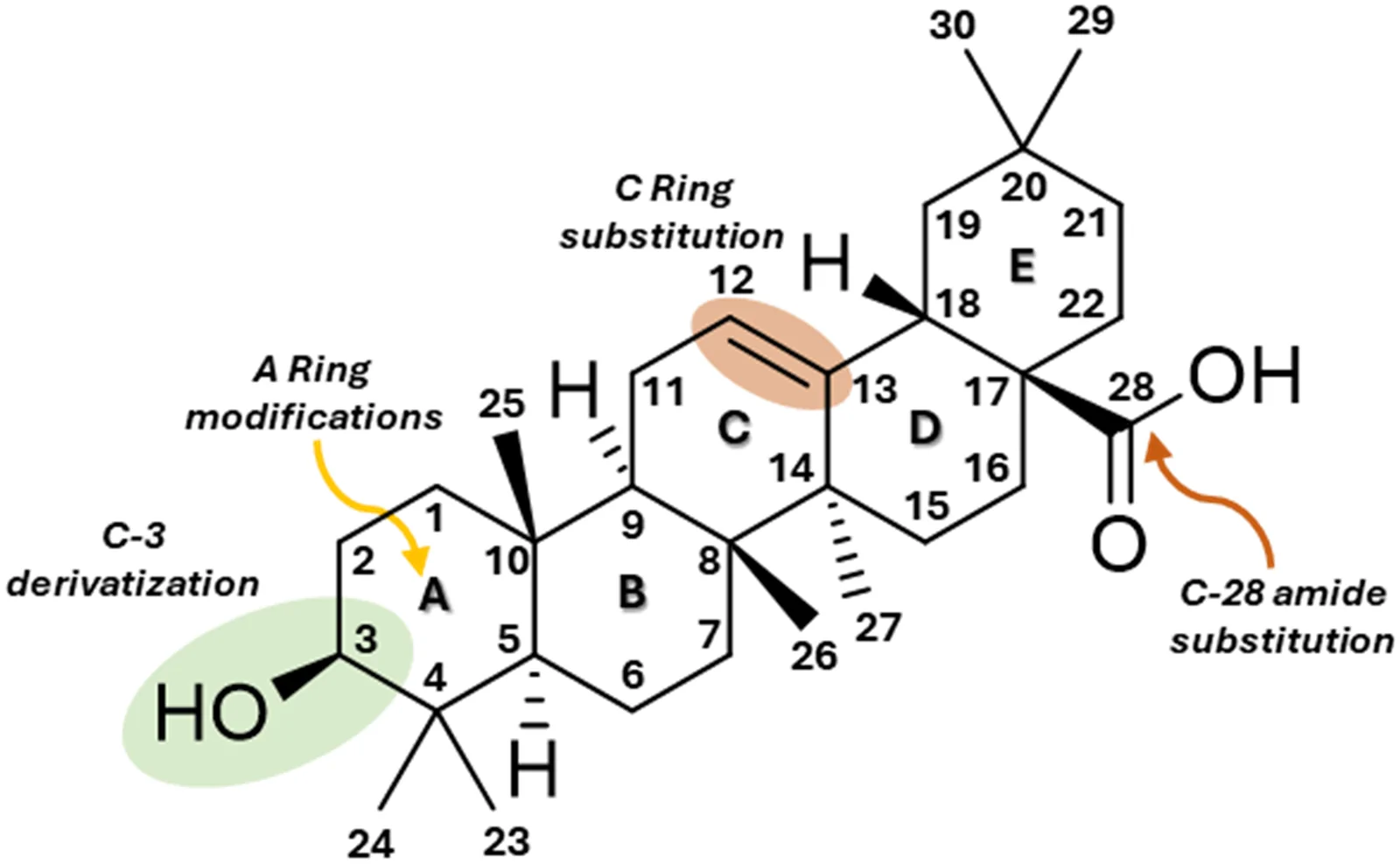
Sites of chemical modifications on oleanolic acid scaffold.

## 2. Results and discussion

To investigate the structure–activity relationship and explore the influence of the double bond on its 3D structure and biological activity, we selected the pentacyclic triterpene δ-oleanolic acid (OA, 1), which is present in a wide range of medicinal plants and the human diet. Since substitution at C-3 and structural changes in the A-ring significantly affect molecular conformation and biological activity, additional analogues featuring C-3 substitution and A-ring rearrangements were synthesized (Fig. 1).

### 2.1 Chemistry

The synthesis of the target derivatives of oleanolic acid, previously isolated from olive leaves, is outlined in Schemes 1 and 2. The isomeric conversion of olean-12-ene skeleton to olean-13(18)-ene skeleton was carried out using the heterogeneous solid–acid catalyst montmorillonite K10 (mont K10).^34^ To control the reaction and prevent the formation of complex mixtures, OA was first esterified. Specifically, treatment of OA with benzyl bromide and potassium carbonate in DMF produced the corresponding benzyl ester 2. Subsequent reaction with mont K10 in DCM smoothly afforded the isomerized benzyl δ-oleanolate (3), which was then subjected to catalytic hydrogenation to yield δ-oleanolic acid (4) (Scheme 1), the structure of which was confirmed by comparison of its MS/HRMS and ^1^H and ^13^C NMR spectra with those reported in the literature.^35^

**Scheme 1:**
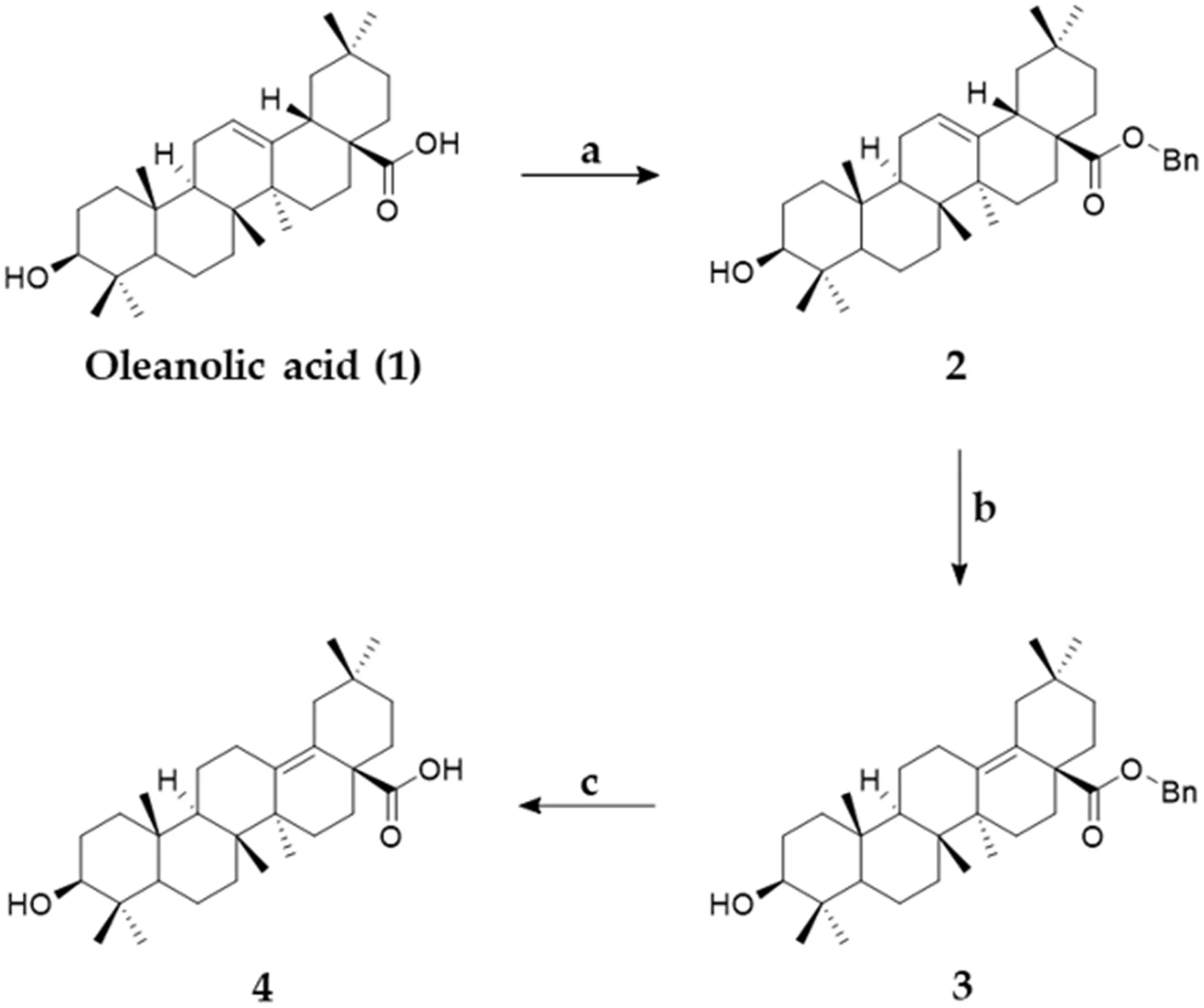
Reagents and conditions: (a) benzyl bromide, K_2_CO_3_, DMF, (b) K-Mont, DCM, (c) H_2_, Pd/C.

**Scheme 2:**
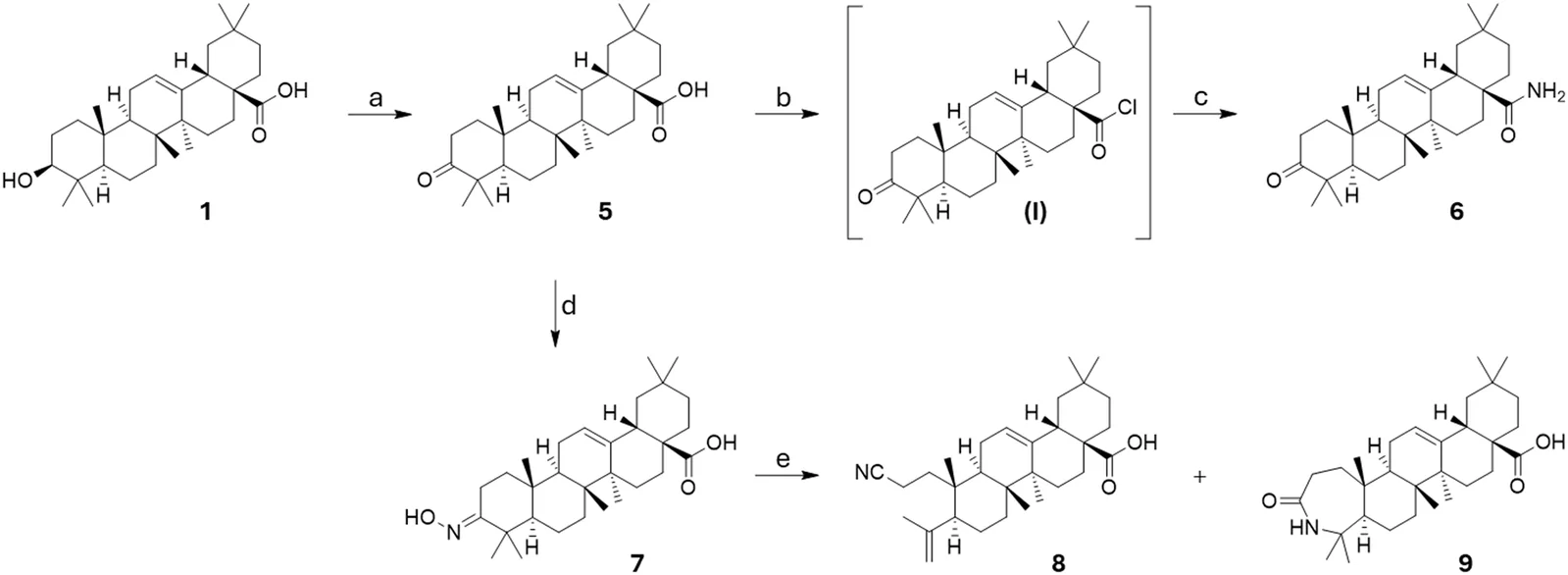
Reagents and conditions: (a) Jones reagent, acetone/DCM, (b) (COCl)_2_, DCM, (c) NH_3_, THF, (d) NH_2_OH HCl, abs. EtOH, (e) SOCl_2_, THF.

Oleanonic acid (5) was prepared *via* the oxidation of 1 with Jones reagent (Scheme 2).^36^ Treatment of 5 with oxalyl chloride afforded the acyl chloride derivative intermediate, which, upon reaction with ammonia, afforded the primary amide 6 in high yield. Its structure was verified by comparing the obtained MS/HRMS and ^1^H and ^13^C NMR spectra with those reported in the literature^37^ (Scheme 2). Furthermore, the oxime 7 was synthesized by reacting hydroxylamine hydrochloride with oleanonic acid in ethanol in the presence of sodium acetate.^38^ The spectral data of the obtained product (7) were also consistent with the literature.^39–41^ Subsequent treatment of oxime 7 with thionyl chloride in THF enabled the modification of ring A, resulting in the A-ring-cleaved derivative 8 as well as the lactam derivative 9. These products were formed *via* the abnormal and classical Beckmann rearrangements, respectively^42^ (Scheme 2). The activity of these two contrasting A-ring outcomes – a flexible, ring-opened nitrile (8) and a conformationally restricted, ring-expanded lactam (9) – was evaluated at both receptors, TGR5 and RORγ. This was done to investigate the effect of A-ring cleavage *versus* expansion and the concomitant introduction of a polar nitrile or lactam nitrogen on activity. Compound 9 was inactive at both receptors, whereas compound 8 retained activity. This indicates that the flexible ring-opened nitrile is compatible with dual-receptor engagement, whereas the more rigid ring-expanded lactam is not. As detailed in section 2.3, docking and MD analyses show that the nitrile and oxime groups of 8 and 7, respectively, engage the RORγ agonist-lock residue H479 directly, unlike 1 and 5, providing a structural rationale for the retained activity of 8 relative to the inactive 9.

### 2.2 Biological evaluation

Pentacyclic triterpenoids were evaluated for their ability to modulate the GPCR TGR5 and the nuclear receptor RORγ. To assess potential agonistic and inverse agonistic properties, respectively, cell-based luciferase reporter gene assays were employed. Initial screening experiments were followed by concentration–response analyses to characterize the potency and efficacy of active compounds.

#### 2.2.1 TGR5 agonistic activity of pentacyclic triterpenoids

To investigate the bioactivity of pentacyclic triterpenoids as TGR5 agonists, a CRE–luciferase reporter gene assay was performed. Initial screening at 10 μM (3 μM for compound 4 due to solubility issues) identified 1, 5, 7, and 8 as active TGR5 agonists (Fig. 2). Among these, 1 (oleanolic acid) and 5 exhibited the highest efficacy, whereas 1 and 8 displayed the highest potency (lowest EC_50_ values; Table 1), indicating strong activation of TGR5-mediated signalling pathways. No agonistic effects were detected in non-TGR5-expressing HEK EPAC cells, confirming receptor-dependent activity.

**Fig. 2:**
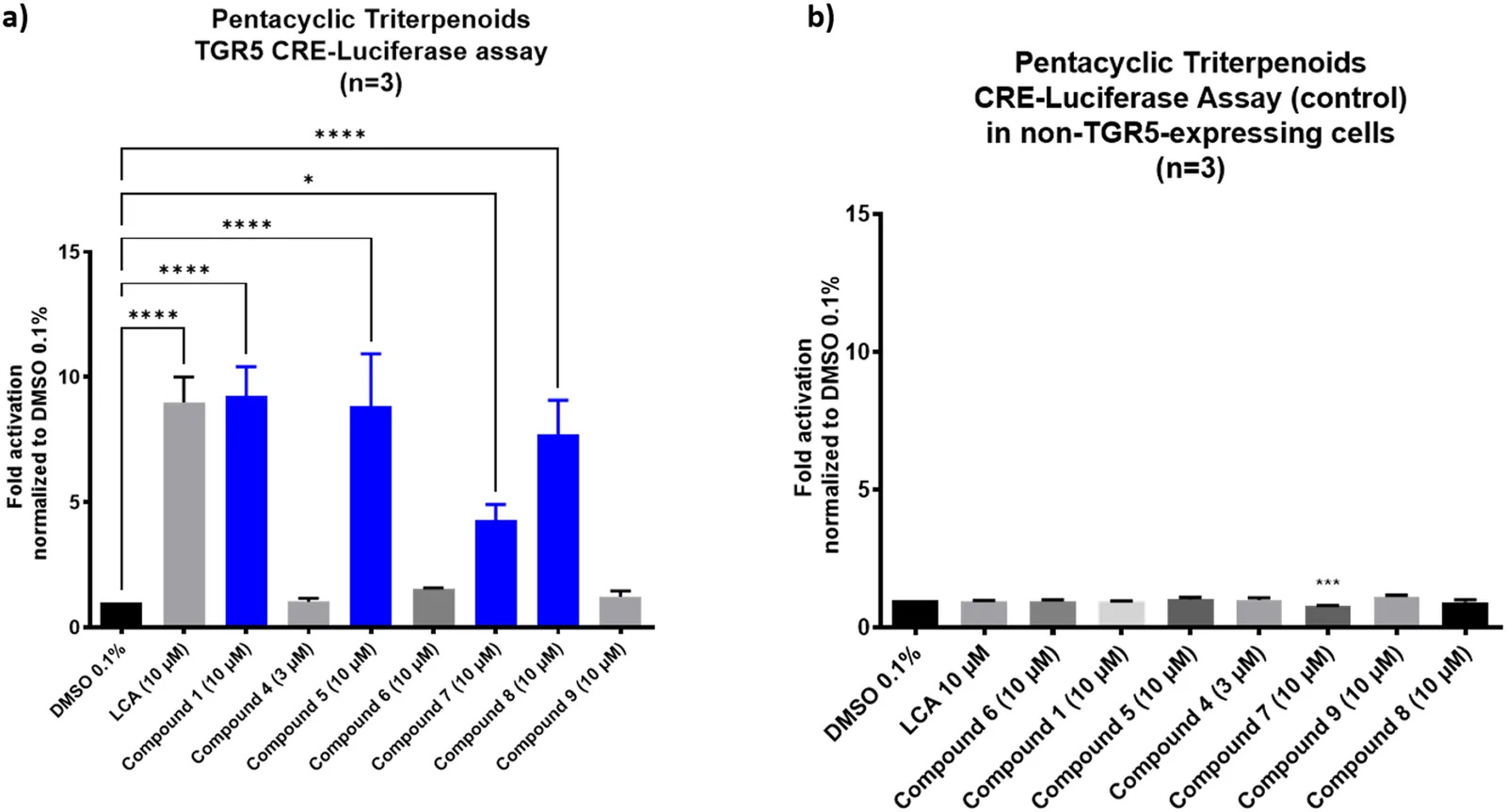
Screening of pentacyclic triterpenoids for TGR5 agonistic activity in CRE–luciferase reporter gene assays. a) A CRE luciferase reporter gene assay was performed in HEK EPAC cells expressing TGR5. b) No agonistic effects were detectable in non-TGR5 expressing HEK EPAC cells in CRE–luciferase assays. Note: in these non-TGR5-expressing cells, compound 7 significantly decreased the basal luciferase signal; compound 4 was tested at 3 μM concentration due to solubility issues. Bar graphs represent the mean ± SD of three biological replicates (*n* = 3) measured in technical quadruplicate. Statistical significance was assessed by one-way ANOVA followed by Dunnett's post hoc test (****p ≤ 0.0001; *p ≤ 0.05; no indication, *p* > 0.05 *vs.* vehicle control).

**Table 1:** Summary of pharmacological activity of selected pentacyclic triterpenoids

Compound	TGR5 EC_50_ (μM)	TGR5 *E*_max_ (fold)	RORγ IC_50_ (μM)	RORγ *I*_max_ (%)
1	1.0	11.18	6.5	51
5	2.5	10.71	—	—
7	15.0*	4.8	14.4	41
8	1.4	9.9	n.d.	39

To further characterize their pharmacological profiles, concentration–response studies were conducted for the active compounds (Fig. 3). All four compounds displayed concentration-dependent activation of TGR5, confirming their TGR5 agonistic activity. Compound 1 showed the highest potency (EC_50_ ≈ 1 μM), followed by 8 and 5, whereas 7 exhibited comparatively weak activity (Table 1). The activity of oleanolic acid (1) and the closely related oleanonic acid (5) is consistent with previous reports,^43^ thereby validating the assay system.

**Fig. 3:**
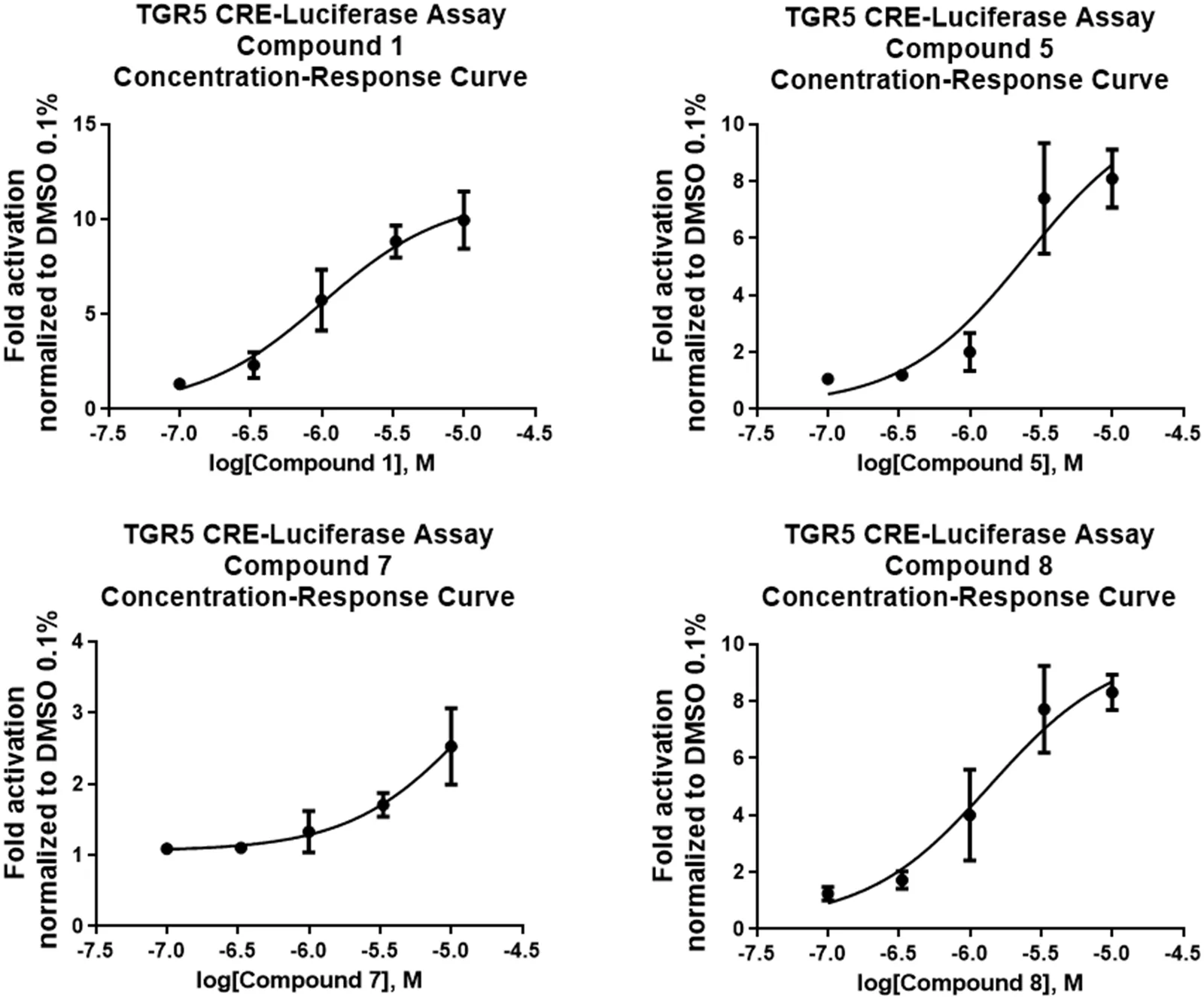
Pentacyclic triterpenoids 1, 5, 7 and 8 concentration-dependently act as TGR5 agonists in CRE–luciferase assays. The panels show concentration–response curves for the indicated compounds in CRE–luciferase assays in TGR5-expressing cells. Data were fitted using a three-parameter logistic nonlinear regression with the Hill slope fixed at −1.0. Each data point represents the mean ± SD of at least three independent biological replicates (*n* ≥ 3), measured in technical quadruplicate.

Notably, derivatives 7 and 8 retained TGR5 agonistic activity despite substantial structural modification of the oleanane scaffold. Of these compounds, only 8 is a newly synthesized derivative, while the known oleanonic acid oxime 7, was evaluated against TGR5 for the first time in this study. In particular, compound 8, a ring-opened derivative, displayed potency (EC_50_ ≈ 1.4 μM), and efficacy comparable to that of oleanolic acid (1), suggesting that structural integrity of this ring system is not strictly required for TGR5 activation. Together, these findings highlight a notable degree of structural tolerance within the triterpenoid scaffold and identify ring-opened derivatives as promising templates for further optimization of TGR5 agonists.

#### 2.2.2 RORγ inverse agonistic activity

To extend the pharmacological characterization of these compounds, their activity on the nuclear receptor RORγ was evaluated using a Gal4–RORγ luciferase reporter assay. Screening at 10 μM (3 μM for compound 4 due to solubility issues) revealed that 1 (oleanolic acid), 7, and 8 act as inverse agonists of RORγ (Fig. 4).

**Fig. 4:**
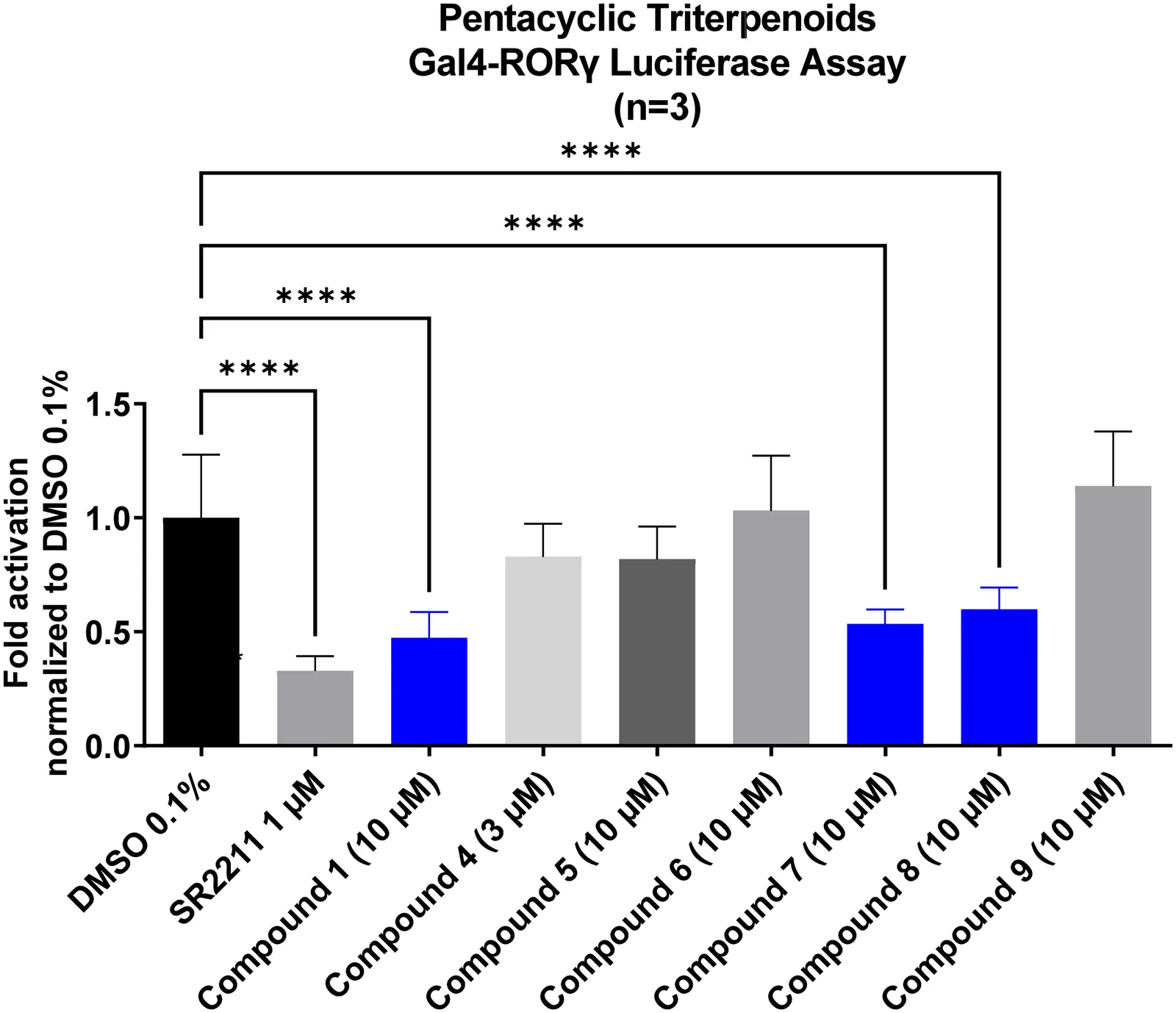
Screening of pentacyclic triterpenoids against RORγ. Pentacyclic triterpenoids were evaluated for inverse agonistic activity on RORγ using a cell-based Gal4–RORγ luciferase reporter assay at 10 μM (3 μM for compound 4 due to solubility issues). The known RORγ inverse agonist SR2211 (1 μM) served as a positive control. Luciferase-derived luminescence signals were normalized to eGFP fluorescence and expressed as fold activation relative to vehicle control. Data are shown as mean ± SD from three independent biological replicates (*n* = 3), each measured in technical quadruplicate. Statistical significance was assessed by one-way ANOVA followed by Dunnett's post hoc test (****p ≤ 0.0001; no indication, *p* > 0.05 *vs.* vehicle control).

Subsequent concentration–response analyses confirmed concentration-dependent inverse agonistic activity for all three compounds (Fig. 5). The reference compound oleanolic acid (1) showed an IC_50_ value consistent with literature reports,^44^ validating the assay system. Compound 7 displayed lower potency than 1, whereas compound 8 produced a concentration-dependent inverse agonistic response but did not allow reliable determination of an IC_50_ value under the conditions tested (Table 1). Nevertheless, both compounds retained pronounced inverse agonistic efficacy, with compound 8 reaching a maximal inhibition of 39%, indicating that RORγ modulation is maintained despite substantial scaffold rearrangement. Importantly, compounds 1, 7, and 8 exhibited activities on both TGR5 and RORγ, supporting their characterization as dual-target modulators at the level of reporter gene assays. Among these, oleanolic acid (1) displayed the most favourable overall potency profile across both targets. In contrast, derivatives 7 and 8 demonstrate that significant structural modification, including ring opening in compound 8, remains compatible with combined TGR5 agonistic and RORγ inverse agonistic activity.

**Fig. 5:**
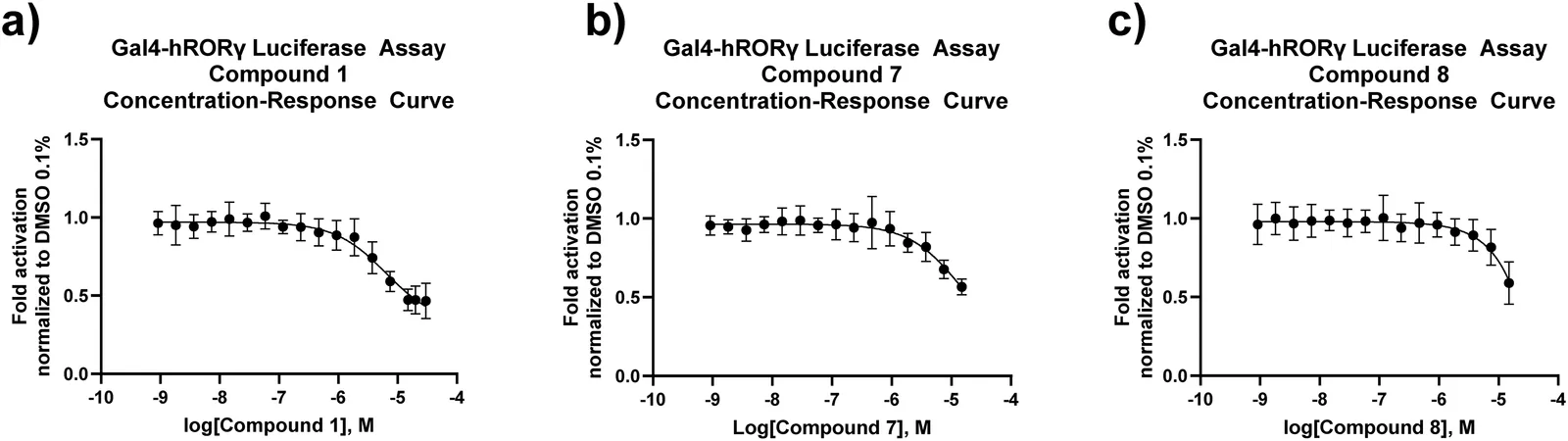
Concentration-dependent activity of the pentacyclic triterpenoids 1 (a), 7 (b), and 8 (c) against the nuclear receptor RORγ. The panels show concentration–response curves for the indicated compounds in a Gal4–RORγ reporter gene assay. Data were fitted using a three-parameter logistic nonlinear regression with the Hill slope fixed at −1.0. Each data point represents the mean ± SD of three independent biological replicates (*n* = 3), measured in technical quadruplicates.

To exclude the possibility that the observed inhibition resulted from nonspecific cytotoxicity, compounds 1, 7, and 8 were evaluated in a resazurin conversion assay under the same treatment conditions as those used in the reporter gene assays (18 h, HEK293 cells).^28^ Relative cell viability remained ≥70% of the vehicle control at all concentrations tested, including the highest, thereby fulfilling the acceptance criterion commonly applied in accordance with ISO 10993-5 (Fig. S1).^45^ Consistently, luciferase activity in the Gal4–RORγ assay was normalised to co-transfected eGFP, which compensates for changes in overall cell number and in general transcriptional and translational activity.

Moreover, the same compounds elicited concentration-dependent increases in luciferase signal of up to 11-fold in the CRE–luciferase assay over a comparable concentration range, an effect that is incompatible with a general cytotoxic mechanism. Taken together, these observations indicate that the RORγ inverse agonistic activity of 1, 7, and 8 reflects specific receptor modulation rather than a mere decrease in cell viability.

Taken together, these findings identify oleanane-derived triterpenoid derivatives as a promising scaffold class for dual TGR5/RORγ modulation and provide initial structure–activity relationship insights for the development of multitarget-directed ligands. Further studies in more complex biological systems will be required to assess the functional consequences of this dual pharmacological profile.

### 2.3 *In silico* modeling

Molecular docking results obtained with the software Glide for PDB structure 7CFN suggest that the pentacyclic ring systems of 1, 5, 7, and 8 bind to TGR5 in a consistent manner, with ring A oriented inside the orthosteric binding site, likely to interact with the residue Y240 (Fig. S2). To further refine the binding poses and calculate binding free energy, MD simulations were conducted (Fig. 6).

**Fig. 6:**
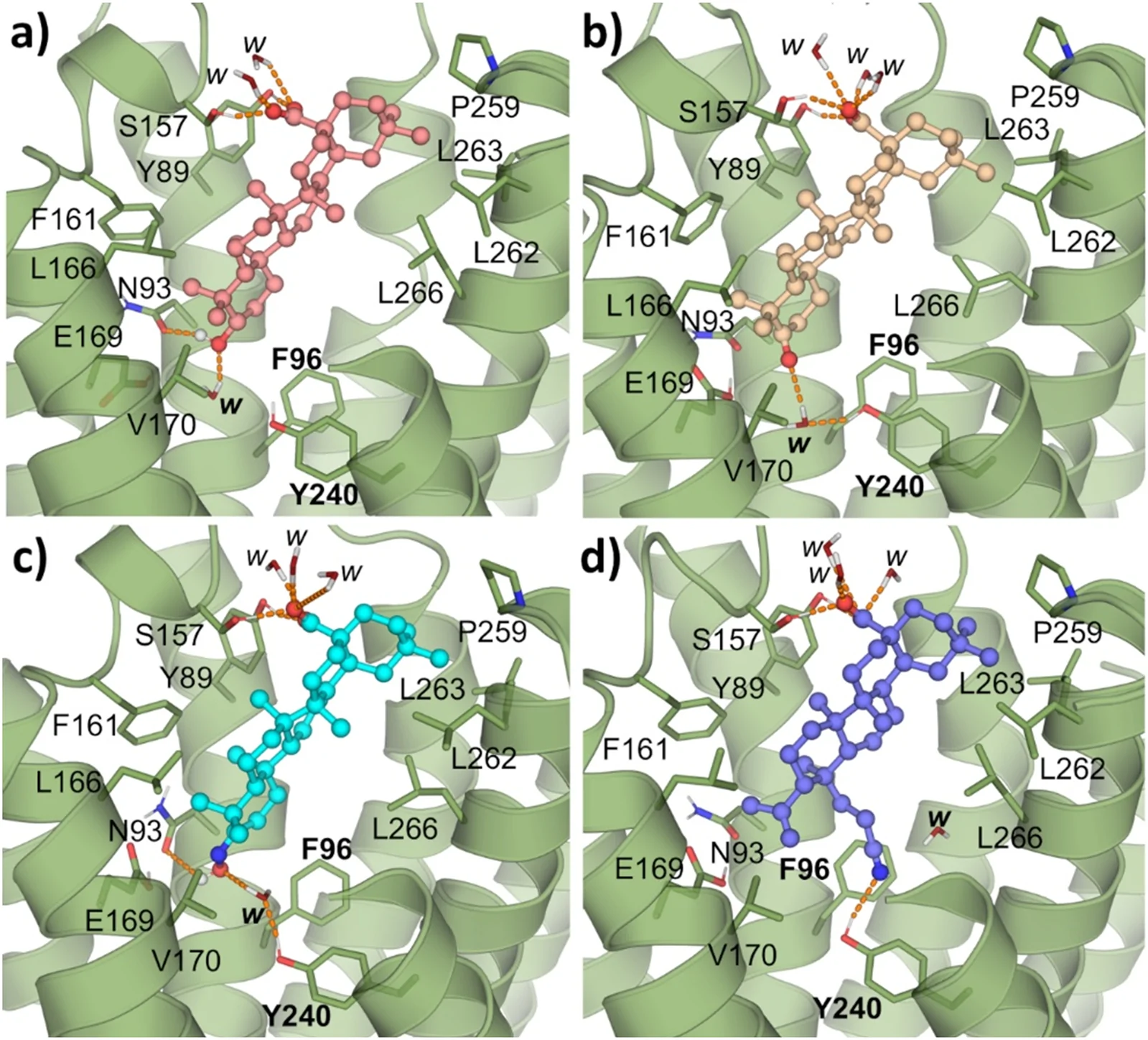
Structural representation of TGR5 in complex with a) 1; b) 5; c) 7; and d) 8. The depicted structures were obtained from clustering analyses of the corresponding 20 ns replica trajectories for each system. Ligands are shown in ball-and-stick representation, while key active-site residues are displayed as sticks and labelled accordingly. Residues F96 and Y240, which participate in stacking interactions, are highlighted in bold. Water molecules interacting with the ligand's carboxylate group or buried at the interface are represented as sticks and labelled *w* or ***w***, respectively. H-bond interactions are depicted as orange dashed lines.

Despite the relatively small RMSD values observed along the 500 ns trajectories with respect to the initial docking poses (∼2 Å at the end of the simulations; Fig. S3), comparison of these poses with representative MD-derived structures revealed two principal structural changes. First, the buried polar oxygens of the ligand shifted away from Y240 and toward residues N93 and E169. Second, the ligand carboxylate groups and residues Y89 and S157 at the pocket entrance underwent reorganization to optimize the polar interaction network in this region (compare Fig. S2 and 6).

Hydrogen bond (H-bond) analysis revealed persistent interactions at the pocket entrance between the carboxylate oxygens of all four compounds and the donor groups of Y89(OH) and S157(OG). In contrast, direct H-bonding with Y240(OH) was less prevalent and observed only in complexes comprising 7 and 8 (Table S1).

Interestingly, trajectory analyses also identified buried water molecules in the TGR5 complexes with 5 and 7. In both systems, water molecules diffused from the bulk solvent into the binding cavity and remained near the ligands' buried carbonyl/hydroxyl oxygen atoms for substantial portions of the simulations (Fig. S4), supporting a role for water-mediated interactions in ligand stabilization. Water bridge analysis further showed interactions between the buried carbonyl oxygen of 5 and Y240(OH)/E169(OE1) at the bottom of the pocket (Table S2). In parallel, the largely solvent-exposed carboxylate oxygens of all analysed ligands formed multiple water bridges with S157 and other residues located at the pocket entrance (Table S2).

Based on these observations, twelve 20 ns replicate MD simulations were initiated from representative conformations extracted from the productive 500 ns trajectories of each TGR5 complex for subsequent molecular mechanics generalized-born surface area (MM-GBSA) binding free energy calculations. The buried water molecules identified in the complexes with 5 and 7 were retained and manually incorporated into the remaining complexes through structural superposition.

The MM-GBSA calculations performed after removing all water molecules from the 20 ns trajectories (Table S3) showed poor agreement with the experimental SAR data (Table S3). In particular, these calculations incorrectly predicted 5 as the least potent and 8 as the most potent ligand in the series. In contrast, the Nwat-MMGBSA free energy calculations, in which the interfacial buried water molecule was retained as part of the receptor (Table 2), reproduced the experimental activity trend, except that 5 was predicted to bind TGR5 slightly more strongly than 8. Overall, these results indicate that inclusion of the buried water substantially improves the correlation between the calculated effective binding free energies (Δ*G*_eff_) and the experimental SAR.

**Table 2:** Nwat-MMGBSA free energy calculations TGR5 in complex with the analyzed compounds^a^

Compound	Δ*E*_vdW_^b^	Δ*E*_el_^c^	Δ*E*_GB_^d^	Δ*E*_surf_^e^	Δ*G*_eff_^f^	ΔΔ*G*^g^
1	−56.38 (0.16)^h^	−178.23 (0.60)	175.82 (0.40)	−7.13 (0.01)	−65.93 (0.46)	—
5	−55.38 (0.11)	−179.03 (0.67)	176.53 (0.70)	−7.13 (0.01)	−65.00 (0.23)	0.93 (0.51)
7	−56.41 (0.29)	−168.54 (0.67)	169.03 (0.43)	−7.32 (0.02)	−63.24 (0.30)	2.69 (0.55)
8	−56.26 (0.29)	−178.43 (0.65)	177.44 (0.71)	−7.57 (0.04)	−64.82 (0.64)	1.11 (0.79)

a All values are expressed in kcal mol^−1^.

b van der Waals contribution.

c Electrostatic contribution.

d Polar solvation free energy calculated using the generalized Born model GB-neck.

e Nonpolar solvation contribution estimated from the solvent-accessible surface area.

f Effective binding free energy, calculated as the sum of the previous energy components.

g Relative binding free energy with respect to compound 1.

h Values in parentheses correspond to the SEM.

Notably, the stabilizing contribution of the retained water molecule was estimated by computing the difference in Δ*G*_eff_ obtained from calculations with and without the buried water as part of the receptor (Tables 2 and S3, respectively).^46^ This analysis showed that the water contribution was more pronounced for 1 and 5 (−2.50 ± 0.51 and −3.70 ± 0.34 kcal mol^−1^, respectively) than for 7 and 8 (−0.49 ± 0.70 and −0.34 ± 0.44 kcal mol^−1^, respectively), for which it was negligible within the estimated error. These results support the importance of water-mediated interactions within the binding cavity as key determinants of the observed SAR for this series of compounds.

Docking results for the RORγ crystal structure 6J3N suggested consistent binding modes for 1, 5, 7, and 8, closely resembling that of the co-crystallized ligand ursonic acid (Fig. S5). Ligand RMSD analyses of the productive 500 ns MD trajectories further indicated that all compounds remained stably bound throughout the simulations, maintaining conformations similar to their initial docking poses, with average RMSD values below 2 Å (Fig. S6). Comparison of the docking poses with the representative MD-derived structures revealed structural rearrangements in two main regions of the binding site. At the pocket entrance, the ligand's carboxylate groups and H323 reoriented to optimize polar interactions. At the bottom of the cavity, adjustments involving H479 and the buried ligand polar groups were also observed. These rearrangements likely contribute to the water-mediated interactions and direct H-bonds discussed below (compare Fig. S5 and 7).

**Fig. 7:**
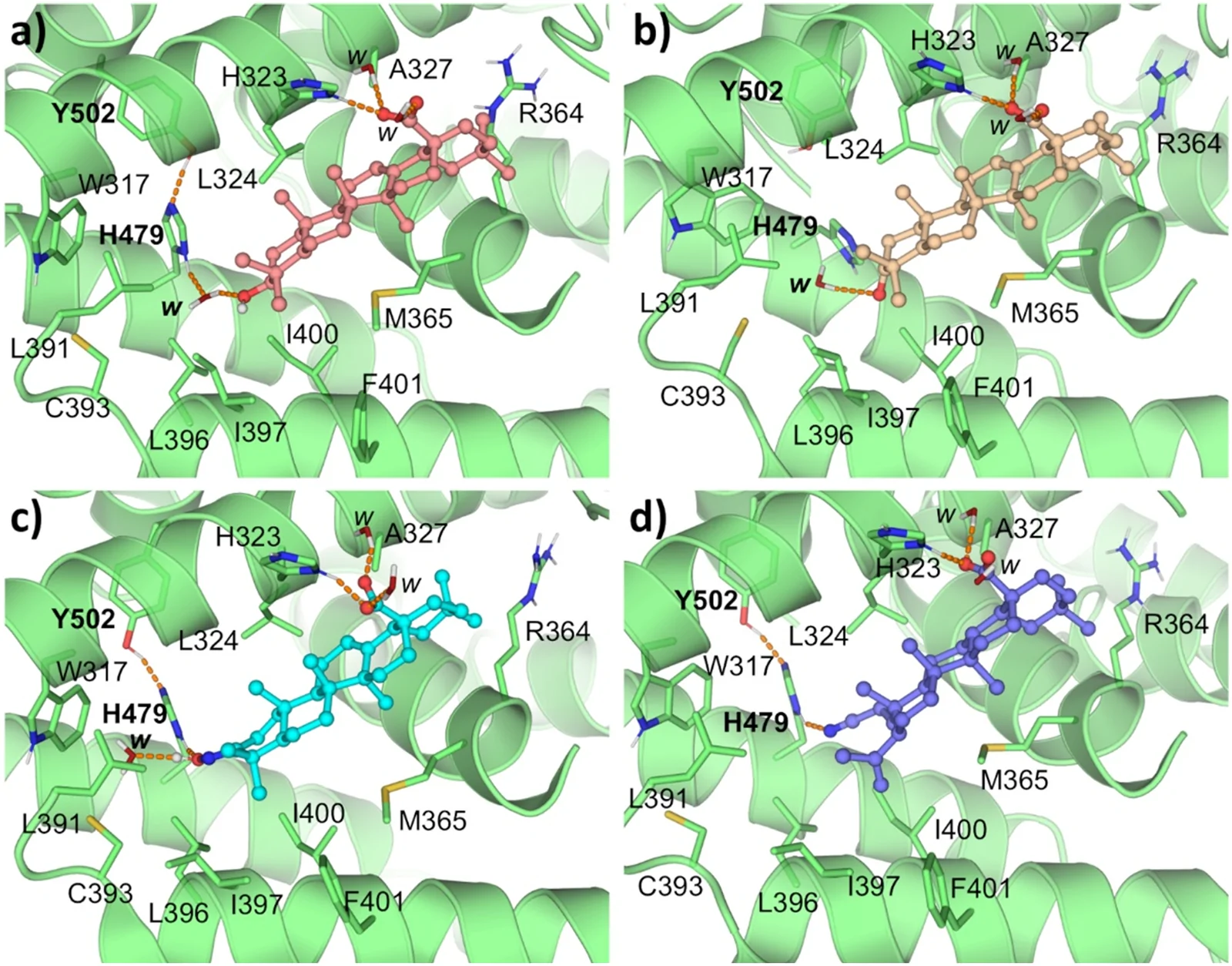
Structural representation of RORγ in complex with a) 1; b) 5; c) 7; and d) 8. The depicted structures were obtained from clustering analyses of the corresponding 20 ns replicate trajectories for each system. Ligands are shown in ball-and-stick representation, while key active-site residues are displayed as sticks and labelled accordingly. Residues Y502 and H479, which form the agonist lock, are highlighted in bold. Water molecules interacting with the ligand carboxylate groups or buried within the binding interface are represented as sticks and labelled as *w* or ***w***, respectively. H-bond interactions are depicted as orange dashed lines.

H-bond analysis showed persistent interactions between the carboxylate oxygens of all four compounds and H323(ND1) (Table S4). Compounds 7 and 8 additionally formed direct H-bonds with H479(ND1) through their hydroxyl and cyano groups, respectively. These interactions are particularly noteworthy, as H479 is part of the agonist lock in RORγ, suggesting that direct engagement of this residue by compounds 7 and 8 may contribute to their inverse agonistic activity.

Water bridge analysis identified water-mediated interactions at two distinct regions of the binding site (Table S5). At the pocket entrance, the carboxylate groups of all four compounds formed stable H-bonds and salt bridges with the positively-charged imidazole ring of H323. At the bottom of the cavity, 1 and 5 formed water bridges with H479 through their hydroxyl and carbonyl oxygens, respectively. More broadly, buried water molecules were found in close proximity to the ligand buried polar groups in all RORγ complexes except 8 for substantial portions of the 500 ns trajectories (Fig. S7), suggesting a general role for water-mediated interactions in ligand stabilization analogous to that observed in the TGR5 complexes.

Again, multiple 20 ns replicate MD simulations were initiated from representative conformations of the RORγ complexes extracted from the productive 500 ns trajectories for subsequent free energy calculations. As in the TGR5 systems, MM-GBSA calculations performed after stripping all water molecules from the trajectories failed to reproduce the experimental SAR, ranking 8 as the most potent RORγ inhibitor and placing 1 second, in contrast to the observed IC_50_ values (Table 1). Retaining the conserved buried water molecule as part of the receptor in the Nwat-MMGBSA calculations corrected this trend, yielding ΔΔ*G* values in better agreement with the experimental data, with 1 ranked as the most potent inhibitor, followed by 7 and 8 with similar predicted affinities, and 5 correctly identified as the least potent in the series (Table 3).

**Table 3:** Nwat-MMGBSA free energy calculations RORγ in complex with the analyzed compounds^a^

Compound	Δ*E*_vdW_^b^	Δ*E*_el_^c^	Δ*E*_GB_^d^	Δ*E*_surf_^e^	Δ*G*_eff_^f^	ΔΔ*G*^g^
1	−57.56 (0.13)^h^	−97.97 (1.37)	108.62 (1.19)	−7.42 (0.02)	−54.34 (0.27)	—
5	−56.05 (0.36)	−82.58 (2.82)	94.40 (2.78)	−6.81 (0.06)	−51.05 (0.34)	3.29 (0.43)
7	−56.07 (0.11)	−93.16 (1.73)	102.62 (1.50)	−6.96 (0.02)	−53.58 (0.35)	0.76 (0.44)
8	−58.64 (0.14)	−94.80 (1.00)	107.24 (0.85)	−7.55 (0.01)	−53.75 (0.32)	0.59 (0.42)

a All values are expressed in kcal mol^−1^.

b van der Waals contribution.

c Electrostatic contribution.

d Polar solvation free energy calculated using the generalized Born model GB-neck.

e Nonpolar solvation contribution estimated from the solvent-accessible surface area.

f Effective binding free energy, calculated as the sum of the previous energy components.

g Relative binding free energy with respect to 1.

h Values in parentheses correspond to the SEM.

Finally, the stabilizing contribution of the retained water molecule was estimated by computing the difference in Δ*G*_eff_ between the two calculation protocols. This analysis revealed a meaningful water contribution for 1, 7, and 5 (−2.41 ± 0.39, −3.47 ± 0.47, and −1.55 ± 0.64 kcal mol^−1^, respectively). For 8, the contribution was negligible within the estimated error (−0.61 ± 0.43 kcal mol^−1^), in line with the absence of buried water molecules observed in its MD trajectories (Fig. S7).

## 3. Conclusions

The present study demonstrates that structural modification of the oleanolic acid scaffold provides access to bioactive triterpenoids capable of modulating the GPCR TGR5 and the nuclear receptor RORγ. Systematic variation of the triterpenoid framework, including alterations of the double bond as well as targeted modulation of the A-ring, provided valuable structure–activity relationship insights and highlighted the sensitivity of receptor activity to subtle conformational changes within the scaffold.

Biological evaluation identified several compounds with pronounced TGR5 agonistic activity. Compounds 1, 5, 7, and 8 induced concentration-dependent receptor activation in CRE–luciferase reporter assays, with oleanolic acid (1) and the newly identified seco-derivative 8 displaying the most favourable potency and efficacy profiles. While the activity of 1 and the closely related derivative 5 is consistent with previous reports, compounds 7 and particularly 8 demonstrate that substantial structural rearrangement, including ring opening, remains compatible with efficient TGR5 activation. These findings reveal a notable degree of structural flexibility within the triterpenoid scaffold and identify ring-opened derivatives as promising templates for further optimization.

Molecular modelling studies provided structural support for these findings, showing that all four compounds adopt similar binding modes within the TGR5 orthosteric pocket. Nwat-MMGBSA calculations ranked 1 as the ligand with the most favourable binding affinity, followed by 5, 8, and 7, in reasonable agreement with the experimental activity data. In contrast, standard MM-GBSA calculations failed to reproduce the experimental activity trend. The improved performance of Nwat-MMGBSA further highlighted the contribution of a conserved buried water molecule to ligand stabilization, particularly for 1 and 5, underscoring the importance of interfacial water molecules in explaining the SAR of this series.

In parallel, selected compounds also displayed inverse agonistic activity toward RORγ, indicating their capacity to interfere with transcriptional programs associated with pro-inflammatory responses. Compounds 7 and 8 retained pronounced inverse agonistic efficacy despite lower potency compared with oleanolic acid (1), indicating that substantial scaffold diversification remains compatible with RORγ modulation. Molecular modelling studies showed that all four compounds adopt binding modes similar to those of the co-crystallized inverse agonist, ursonic acid, establishing direct and water-mediated interactions with key residues at the pocket entrance and the bottom of the cavity. Compounds 7 and 8 formed direct H-bonds with H479, a residue of the RORγ agonist lock, whereas 1 and 5 interacted indirectly with this residue through water bridges. These results suggest that both direct and water-mediated engagement of the agonist lock may contribute to the observed inverse agonistic activity. Notably, water-mediated interactions between ligand buried polar groups and agonist-lock residues have also been reported in the RORγ crystal structure 3L0L,^47^ supporting the binding modes identified here for 1 and 5. Consistent with the experimental activity data, Nwat-MMGBSA calculations ranked 1 as the most potent inhibitor, followed by 7 and 8 with similar predicted affinities, and 5 as the least potent, further supporting the stabilizing role of conserved buried water molecules in these complexes.

Importantly, compounds 1, 7, and 8 exhibited activities at both TGR5 and RORγ, supporting their characterization as dual-target modulators at the level of reporter gene assays. Collectively, the results demonstrate that oleanolic acid-derived triterpenoid scaffolds tolerate substantial structural diversification while maintaining combined TGR5 agonistic and RORγ inverse agonistic activity. In particular, the identification of the ring-opened seco-derivative 8 as a potent TGR5 agonist with retained RORγ activity represents a notable finding of this study and expands the accessible chemical space for multitarget triterpenoid ligands.

Overall, these findings provide a basis for further developing triterpenoid-based dual modulators that target TGR5 and RORγ. Future studies should focus on refining structure–activity relationships, elucidating the mechanistic basis of receptor modulation and evaluating the pharmacological and functional consequences of dual activity in more advanced biological systems. The direct hydrogen bond formed by compounds 7 and 8 with the RORγ agonist-lock residue H479, in contrast to the water-mediated engagement of this residue by compounds 1 and 5, offers a concrete structure-based design strategy for the next generation of analogues. Additional H479-directed polar substituents at the A-ring, or the extension of this strategy to other pocket-entrance vectors, may improve the potency of ring-opened, more soluble derivatives without compromising dual TGR5/RORγ activity. Future work will also examine whether the conserved buried water molecule, as highlighted by the Nwat-MMGBSA calculations, can be displaced or mimicked by design to enhance TGR5 potency.

## 4. Experimental section

### 4.1 Chemistry

#### 4.1.1 Materials and methods

Ethyl acetate (EtOAc), *n*-hexane, methanol (MeOH), dichloromethane (DCM), cyclohexane (*c*-hexane), isopropanol and tetrahydrofuran (THF) used for extraction, purification, and as reaction solvents were of analytical grade (Fisher Scientific, Loughborough, Belgium), while water (H_2_O) was distilled. For pH adjustment, sodium hydroxide pellets (NaOH^−^ penta CHEMICALS UNITED) and hydrochloric acid (HCl, analytical grade; Fisher Scientific, Loughborough, Belgium) were used. Ethanol (EtOH, Avantor Performance Materials, Poland), acetonitrile (ACN, Avantor Performance Materials, Poland), and H_2_O (Fisher Scientific, Loughborough, Belgium) used as reaction solvent and for preparative high-performance liquid chromatography (prep-HPLC) analysis were of HPLC grade. Reaction progress was monitored by thin-layer chromatography on pre-coated silica gel 60 F254 plates (0.25 mm thickness) from Merck, visualized at 254 and 366 nm (UV lamp). Flash chromatography was performed using Merck silica gel 60A (0.040−0.060 mm) (Merck, Kenilworth, NJ) with the specified solvent system, typically applying gradients of increasing polarity. All commercially available reagents were purchased from Alfa Aesar and used without any further purification. The isolation of MNA was performed using preparative RP-HPLC (Buchi, Pure C-850 FlashPrep) hyphenated to a photo diode array (PDA) detector. All solvent evaporations were performed using a rotary evaporator (Buchi) with a water bath at 40 °C. ^1^H NMR, ^13^C NMR, and 2D spectra were recorded on a Bruker Avance III 600 MHz spectrometer (Bruker Biospin AG, Faellanden, Switzerland) and on a Bruker Avance NEO 400 MHz spectrometer (Bruker) in deuterated solvents (see SI). Chemical shifts are expressed as *δ* values in parts per million (ppm), and the coupling constants (*J*) are given in Hertz (Hz). The signals of ^1^H and ^13^C NMR spectra were unambiguously assigned by using 2D NMR techniques: ^1^H–^1^H COSY, HSQC-DEPT 135, NOESY and HMBC experiments. The signals are reported as follows: (s = singlet, d = doublet, t = triplet, m = multiplet, br = broad). HRMS spectra were obtained on a LTQ-Orbitrap Discovery Mass Spectrometer (Thermo Scientific, Bremen, Germany) and on a Triple TOF 5600+ ABSciex.

#### 4.1.2 Isolation of oleanolic acid (1)

Oleanolic acid (1) was isolated following a previously described procedure (DOI: https://doi.org/10.3390/molecules30132709). Briefly, olive leaves were used as raw material, from which an extract rich in oleanolic and maslinic acid was obtained by supercritical fluid extraction (SFE). Fractionation of the triterpenic acids was subsequently achieved using pH-zone-refining centrifugal partition chromatography (CPC), and 1 was isolated in high yield.

#### 4.1.3 Synthesis of δ-oleanolic acid (4)

A mixture of 1 (250.0 mg, 0.54 mmol), benzyl bromide (4.0 eq., 2.16 mmol, 0.25 mL) and K _2_CO_3_ (3.0 eq., 1.62 mmol, 223.9 mg) in DMF (4.0 mL) was stirred under argon for 3 h. After completion of the reaction, H_2_O and 6 N HCl were added for acidification, followed by extraction with EtOAc. The combined organic layers were washed with H_2_O (3×), dried over anhydrous Na_2_SO_4_, and concentrated under reduced pressure to afford an oily residue, which was purified by column chromatography (silica gel) using 100% DCM as the eluent. The benzyl ester of oleanolic acid (2) was isolated as a white solid (220.0 mg, yield = 76%). Then, 2 was dissolved in 10.0 mL DCM, and 240.0 mg of H-montmorillonite were added. H-montmorillonite was prepared by stirring a mixture of mont K10 (3.0 g) and 1.1 wt% HCl solution (200.0 mL) at 90 °C for 24 h. The mixture was filtered, washed with H_2_O, and dried at 110 °C to give the catalyst as a white powder. The mixture of 2 was stirred at 40 °C for 2 h, filtered, and the solvent was evaporated under reduced pressure. The crude product was purified by column chromatography (100% DCM) to yield the δ-benzyl ester of oleanolic acid (3) (200.0 mg, yield = 91%). Catalytic hydrogenation over 10% Pd/C (20 mg), at 50 psi, in absolute EtOH (30.0 mL), afforded δ-oleanolic acid (4) in 90% yield. The spectroscopic data for the product were consistent with those reported in the literature.^35^

#### 4.1.4 Synthesis of oleanonic acid (5)

Jones reagent (2.0 mL) was added dropwise to a solution of 1 (3.0 g, 6.56 mmol) in acetone/DCM (1 : 1, v/v, 200.0 mL) at 0 °C. Upon completion of the reaction, isopropanol was added to quench any excess of the reagent. The solvent was evaporated, and the oily residue was dissolved in EtOAc and extracted with H_2_O. The organic phase was washed with H_2_O (3×), dried over anhydrous Na_2_SO_4_, and concentrated to dryness to afford 2.8 g of pure oleanonic acid (5) (yield = 94%). The spectroscopic data for the product were consistent with those reported in the literature.^48^

#### 4.1.5 Synthesis of 3-oxoolean-12-en-28-amide (6)

Oxalyl chloride (5.0 eq., 2.10 mmol, 0.18 mL) was added dropwise to a solution of 5 (192.6 mg, 0.42 mmol) in anhydrous DCM (15.0 mL), and the resulting mixture was stirred at room temperature, under argon, for 2 h. After completion of the reaction, DCM was removed under reduced pressure, and the residue was dissolved in dry THF. The solution was cooled to 0 °C, and ammonia solution 24% (20 eq., 8.4 mmol, 0.20 mL) was added. After 10 minutes, the solvent was removed under reduced pressure, and the mixture was extracted with EtOAc and H_2_O. The organic phase was dried over anhydrous Na_2_SO_4_ and concentrated under reduced pressure, affording 190.2 mg of 6 (yield = 99%). The spectroscopic data for the product were consistent with those reported in the literature.^37^

#### 4.1.6 Synthesis of 3-hydroxyimino-olean-12-en-28-oic acid (7)

A mixture of 5 (1.5 g, 3.32 mmol), hydroxylamine hydrochloride (5.0 eq., 16.6 mmol, 1.15 g) and sodium acetate (6.0 eq., 19.92 mmol, 1.63 g) in absolute EtOH (50.0 mL) was stirred under reflux for 2 h. After evaporation of two-thirds of the ethanol, H2O was added, and the solution was left at 4 °C for 12 h, allowing the product to precipitate. The solid was filtered and dried to give oxime 7 as a white solid (1.2 g, yield = 76%). The spectroscopic data for the product were consistent with those reported in the literature.^49^

#### 4.1.7 Synthesis of 3-cyano-3,4-seco-4-ylidenolean-12-en-28-oic acid (8) and 3-oxo-3α-omo-3α-aza-olean-12-en-28-oic acid (9)

Thionyl chloride (6.0 eq., 15.42 mmol, 1.11 mL) was added dropwise to a solution of 7 (1.2 g, 2.57 mmol) in anhydrous THF (10.0 mL), under argon, and the reaction mixture was stirred at room temperature for 3 h. After completion of the reaction, H_2_O and ice were added, and the mixture was stirred for 10 min. The mixture was extracted with EtOAc, and the organic layer was washed with H_2_O (3×), dried over anhydrous Na_2_SO_4_, and concentrated under reduced pressure. The crude residue was purified by column chromatography (silica gel) using a DCM/MeOH elution system. Compound 8 was eluted with 99% DCM (260.0 mg) and 9 with 97% DCM (305.0 mg).

##### Spectroscopic data of 8

^1^H NMR (600 MHz, CDCl_3_) *δ* (ppm) 5.29 (1H, t, *J* = 3.28 Hz, CH-12), 4.89 (1H, brs, CH-23a), 4.65 (1H, brs, CH-23b), 2.84 (1H, dd, *J* = 4.20/14.01 Hz, CH-18), 2.34 (1H, m, CH-2a), 2.24 (1H, m, CH-2b), 1.99 (1H, m, CH-9), 1.90 (1H, dd, *J* = 2.55/12.56 Hz, CH-5), 1.78 (2H, m, CH_2_-22), 1.76 (1H, m, CH-16a), 1.73 (1H, m, CH-11a), 1.72 (3H, s, CH_3_-3), 1.69 (1H, m, CH-15a), 1.65 (2H, m, CH_2_-1), 1.613 (1H, m, CH-19a), 1.610 (2H, m, CH_2_-6), 1.57 (1H, m, CH-16b), 1.44 (1H, m, CH-21a), 1.41 (1H, m, CH-11b), 1.34 (1H, m, CH-7a), 1.28 (1H, m, CH-21b), 1.23 (1H, m, CH-7b), 1.18 (1H, m, CH-19b), 1.15 (3H, s, CH_3_-26), 1.12 (1H, m, CH-15b), 0.93 (3H, s, CH_3_-29), 0.92 (3H, s, CH_3_-24), 0.91 (3H, s, CH_3_-28), 0.81 (3H, s, CH_3_-25). ^13^C NMR (151 MHz, CDCl_3_) *δ* (ppm) 183.17 (C, C-27), 146.96 (C, C-4), 144.00 (C, C-13), 122.15 (CH, C-12), 120.38 (C, C-30), 114.40 (CH_2_, C-23), 50.89 (CH, C-5), 46.72 (C, C-17), 45.93 (CH_2_, C-19), 42.33 (C, C-14), 41.29 (CH, C-18), 39.70 (C, C-10), 39.27 (C, C-8), 38.12 (CH, C-9), 34.60 (CH_2_, C-1), 34.00 (CH_2_, C-7), 33.24 (CH_3_, C-28), 32.57 (CH_2_, C-16), 31.46 (CH_2_, C-21), 30.89 (C, C-20), 27.83 (CH_2_, C-15), 25.97 (CH_3_, C-26), 24.33 (CH_2_, C-11), 23.85 (CH_2_, C-22), 23.73 (CH_3_, C-29), 23.17 (CH_3_, C-3), 23.08 (CH_2_, C-6), 19.24 (CH_3_, C-24), 17.32 (CH_3_, C-25), 11.77 (CH_2_, C-2). LC-ESI-HRMS (−) calcd. for C_30_H_44_NO_2_^−^: *m*/*z* 450.3378, found 450.3375.

##### Spectroscopic data of 9

^1^H NMR (600 MHz, CDCl_3_) *δ* (ppm) 5.30 (1H, t, *J* = 3.31 Hz, CH-12), 2.84 (1H, dd, *J* = 4.09/14.10 Hz, CH-18), 2.52 (1H, m, CH-2a), 2.43 (1H, m, CH-2b), 1.94 (2H, m, CH1-11), 1.77 (1H, m, CH-7a), 1.76 (1H, m, CH-1a), 1.71 (1H, m, CH-15a), 1.68 (1H, m, CH-9), 1.62 (2H, m, CH_2_-22), 1.62 (1H, m, CH-19a), 1.59-1.53 (1H, m, CH-6a), 1.57 (1H, m, CH-7b), 1.44 (1H, m, CH-1b), 1.39 (1H, m, CH-6b), 1.39 (1H, m, CH-5), 1.33 (2H, m, CH_2_-16), 1.29 (3H, s, CH_3_-24), 1.27 (3H, s, CH_3_-23), 1.21 (2H, m, CH_2_-21), 1.16 (1H, m, CH-19b), 1.14 (3H, s, CH_3_-27), 1.11 (3H, s, CH_3_-25), 1.09 (1H, m, CH-15b), 0.93 (3H, s, CH_3_-30), 0.91 (3H, s, CH_3_-29), 0.82 (3H, s, CH_3_-26). ^13^C NMR (151 MHz, CDCl_3_) *δ* (ppm) 181.77 (C, C-28), 177.68 (C, C-3), 143.66 (C, C-13), 122.76 (CH, C-12), 56.95 (CH, C-4), 55.87 (CH, C-5), 47.42 (CH, C-9), 46.71 (C, C-17), 46.02 (CH_2_, C-19), 42.16 (C, C-14), 41.33 (CH, C-18), 41.21 (C, C-10), 39.69 (C, C-8), 37.22 (CH_2_, C-1), 34.68 (CH_3_, C-24), 34.07 (CH_2_, C-21), 33.27 (CH_3_, C-29), 32.73 (CH_2_, C-16), 32.63 (CH_2_, C-7), 31.51 (CH_2_, C-2), 30.90 (C, C-20), 27.87 (CH_2_, C-15), 26.46 (CH_3_, C-23), 25.85 (CH_3_, C-27), 24.07 (CH_2_, C-11), 23.78 (CH_3_-30), 23.19 (CH_2_, C-22), 22.01 (CH_2_, C-6), 17.38 (CH_3_, C-26), 16.54 (CH_3_, C-25). LC-ESI-HRMS (−) calcd. for C_30_H_46_NO_3_^−^: *m*/*z* 468.3483, found 468.3483.

### 4.2 Biology

#### 4.2.1 Materials

Human embryonic kidney 293 (HEK293) cells (Cat. #CRL-1573) were purchased from the American Type Culture Collection (ATCC; Manassas, VA, USA). Dulbecco's modified Eagle medium (DMEM; Cat. #D6546), coenzyme A (CoA; Cat. #C3019), dithiothreitol (DTT; Cat. #D5545), l-glutamine (Cat. #G7513), and penicillin–streptomycin (Cat. #P0781) were obtained from Sigma-Aldrich (St. Louis, MO, USA). Fetal bovine serum (FBS; Cat. #S1810, Batch #S00CN) was purchased from Biowest (Nuaillé, France). The 5× reporter lysis buffer (Cat. #E3971) was obtained from Promega (Fitchburg, WI, USA). Dimethyl sulfoxide (DMSO; Cat. #4720.2), adenosine-5′-triphosphate (ATP; Cat. #HN35.3), ethylenediaminetetraacetic acid (EDTA; Cat. #8043.2), HEPES (Cat. #6763.2), Na_2_HPO_4_ (Cat. #T876.2), and NaCl (Cat. #9265.2) were purchased from Carl Roth GmbH & Co. KG (Karlsruhe, Germany). d-Luciferin sodium salt (BC218; Cat. #s039) was purchased from Synchem UG & Co. KG (Felsberg, Germany). Trypsin (Cat. #27250-0180) was obtained from Thermo Fisher Scientific (Waltham, MA, USA). SR2211 (Cat. #HY-16998) was purchased from MedChemExpress (Monmouth Junction, NJ, USA). Calcium chloride dihydrate (CaCl_2_·2H_2_O; Cat. #15692220) was obtained from Fluka Analytical (St. Gallen, Switzerland). Phenol red-free DMEM (Cat. #17-205-CV) was purchased from Corning (Manassas, VA, USA).

#### 4.2.2 Plasmids

The RORγ–Gal4 construct was kindly provided by Dr. Fabio R. Santori (Center for Molecular Medicine, University of Georgia, Athens, GA, USA). The Gal4-response element tk(MH1000)4×Luc was obtained from Prof. Ronald Evans (Salk Institute for Biological Studies, La Jolla, CA, USA). The eGFP plasmid (pEGFP-N1) was purchased from Clontech (Mountain View, CA, USA).

#### 4.2.3 Cell culture

HEK293 cells were maintained in DMEM with 4.5 g L^−1^ glucose and phenol red containing 10% FBS, 2 mM glutamine, 100 U mL^−1^ penicillin, and 100 μg mL^−1^ streptomycin (“DMEM complete”). Where specified, DMEM containing 4.5 g L^−1^ glucose and phenol red was supplemented with 5% charcoal-stripped FBS (“DMEM stripped”) instead of 10% FBS, while all other components remained unchanged. Cultures were kept at 37 °C with 5% CO_2_ and passaged every 2–3 days. Cells were used only up to passage 30. Cells were detached from culture flasks following incubation with trypsin/EDTA. Cell number and viability were assessed with an automated cell counter (Vi-CELL XR Cell Viability Analyzer, Beckmann, Krefeld, Germany).

#### 4.2.4 Luciferase reporter gene assays

HEK293 cells (8 × 10^6^ per 150 mm dish) were seeded in complete DMEM. For CRE–luciferase assays, TGR5 HEK EPAC cells (stably expressing TGR5 (ref. 50) and an EPAC-based FRET biosensor^51^ as described previously) were used. Transient transfections were performed overnight using the calcium phosphate method with the respective receptor and luciferase reporter plasmids, together with eGFP.^52^ For CRE–luciferase assays, 10 μg of CRE–luciferase reporter was transfected. For Gal4–luciferase assays, a RORγ–Gal4 hybrid construct (ligand-binding domain of RORγ fused to the Gal4 DNA-binding domain) was used with 5 μg RORγ–Gal4, 5 μg Gal4-response element tk(MH1000)4×Luc, and 3 μg pEGFP-N1. The Gal4–RORγ assay was used to read out ligand-dependent transcription driven specifically by the RORγ ligand-binding domain. By replacing the native RORγ DNA-binding domain and response elements with Gal4/UAS, this assay reduces confounding from (i) endogenous proteins that bind or modulate native ROR response elements, (ii) promoter- and chromatin-context effects tied to the full-length receptor, and (iii) cell-specific coactivator/corepressor pools that differentially engage native promoters. In addition, this assay matched the RORγ-LBD used in our structural and simulation work (PDB 6J3N; section 2.3), allowing a direct, mechanism-aligned comparison between docking/MD predictions and cellular activity. After transfection, cells were resuspended in 5% charcoal-stripped FBS-DMEM, reseeded at 5 × 10^4^ cells/well in 96-well plates, and treated with test compounds for 18 h. Cells were lysed in reporter lysis buffer (1×) supplemented with 90 μM CoA and 1 mM DTT. Luminescence and fluorescence were measured on a TECAN Spark Microplate Reader (Tecan Group, Männedorf, Switzerland) after sequential injection of 50 μL ATP solution (3.7 mM ATP, 20 mM tricine, pH 7.8, 21.5 mM MgCl_2_) and 50 μL luciferin solution (0.32 mg mL^−1^d-luciferin, 21 mM tricine, pH 7.8). Luciferase activity was normalized to GFP fluorescence and expressed as fold activation relative to vehicle (0.1% DMSO).

#### 4.2.5 Resazurin conversion assay

For the resazurin conversion assay, HEK293 cells were treated with vehicle control, the cytotoxic positive control (digitonin, 50 μg mL^−1^), or the compounds of interest for 18 h. Subsequently, the treatment medium was removed and replaced with phenol red-free DMEM containing 10 μg mL^−1^ resazurin sodium salt. After incubation for 5 h, fluorescence was measured at an excitation wavelength of 535 nm and an emission wavelength of 590 nm using a TECAN Spark microplate reader. Compounds were considered non-cytotoxic when relative cell viability remained ≥70% of the vehicle control, in accordance with ISO 10993-5.^45^ Stock solutions of all compounds were freshly prepared in DMSO and diluted into the culture medium immediately before treatment (final DMSO concentration 0.1%). The assay was performed in five independent biological experiments (*n* = 5), each with four technical replicates.

#### 4.2.6 Statistical analysis

Luciferase assays were performed independently three times, and the resazurin conversion assay five times, each with four technical replicates per condition. Data analysis and graph preparation were carried out in GraphPad Prism version 9.4 (Dotmatics, Boston, MA, USA). Bioactivity values are presented as mean ± standard deviation unless stated otherwise. Outliers were identified using the ROUT method at *Q* = 10%. To assess statistically significant differences between compound-treated samples and the DMSO vehicle control, a one-way analysis of variance (ANOVA) followed by Dunnett's post hoc test was applied. Statistical significance is reported as follows: ****p ≤ 0.0001, ***p ≤ 0.001, **p ≤ 0.01, *p ≤ 0.05, and no indication for *p* > 0.05. Concentration–response curves were generated using a three-parameter logistic nonlinear regression, with the Hill slope fixed to −1.0. IC_50_ values were derived from the best-fit curves. “Apparent” EC_50_/IC_50_ values are reported when the calculated EC_50_/IC_50_ value fell outside the tested concentration range. *E*_max_ values are equal to the fold activation observed at the highest concentration tested. *I*_max(%)_ values are calculated as follows: *I*_max(%)_ = (1 − Bottom/Top) × 100, where “Top” corresponds to baseline activity at the lowest concentration and “Bottom” to residual activity at the highest concentration (*i.e.*, maximal inhibitory effect).

### 4.3 *In silico* modeling

#### 4.3.1 Molecular docking

Protein structures for TGR5 (PDB: 7CFN, chain R) and RORγ-LBD (PDB: 6J3N) were downloaded from the Protein Data Bank (PDB)^53^ and prepared with the Protein Preparation Wizard^54,55^ (part of the Schrödinger Platform) with default settings, including assigning bond orders, adding hydrogens, removing solvent molecules, optimizing the H-bond network, and minimizing the protein structures. Water molecules located more than 5 Å from the ligand were removed.

In preparation for docking, receptor grids were generated for each protein structure with the Receptor Grid Generation Wizard (part of the Schrödinger Platform). The location and size of the individual grids were defined based on the co-ligand with default settings. For TGR5, the orthosteric site was defined as the binding pocket.

The molecular structures of all compounds were prepared for docking with LigPrep^55^ (part of the Schrödinger Platform) using default settings, employing Epik^56^ (also part of the Schrödinger Platform) to assign ionization states at pH 7 ± 0, and optimizing geometries with the OPLS4 force field.^57^ Docking was performed with Glide^58^ part of the Schrödinger Platform for drug discovery, version 2024-2 in Standard Precision mode (Glide SP). All prepared compounds were docked against the binding pockets. Up to 20 docking poses were stored and inspected for each binding pocket and compound.

#### 4.3.2 Parametrization of ligand atomic charges for MD simulations

The structures of the OL compounds investigated in this study were initially subjected to geometry optimization in vacuum using Gaussian 09 at the B3LYP/6-31G(d,p) level of theory.^59^ The optimized geometries were subsequently employed in single-point energy calculations to obtain the electrostatic potential (ESP) at the Hartree–Fock level with the 6-31G(d) basis set. Atomic partial charges were then derived by fitting the ESP using the RESP procedure implemented in the *antechamber* module of AmberTools24.^60^

#### 4.3.3 MD simulations

The TGR5 and RORγ structures employed in the MD simulations were prepared using the same protonation states assigned during the docking studies. System topologies and coordinates were generated with the *tleap* module of AmberTools24.^60^ Protein parameters were described with the ff19SB force field,^61^ whereas ligand parameters were assigned according to GAFF2.^60^

For the RORγ complexes, the systems were immersed in truncated octahedral boxes containing OPC water molecules and the appropriate number of counterions to ensure electroneutrality, maintaining a minimum distance of 10 Å between the protein surface and the box boundaries. TGR5 systems were instead modelled in an explicit membrane environment composed of 209 lipid molecules (69 CHL, 59 POPC, 36 PSM, 32 POPE, and 13 POPS). The orientation of the receptor within the membrane was obtained from the OPM web server,^62^ and the lipid bilayers were assembled with CHARMM-GUI.^63^ Lipid parameters were drawn from the lipid21 force field.^64^ The resulting protein–membrane systems were solvated with OPC water molecules^65^ and neutralizing ions in rectangular simulation boxes extending at least 15 Å from the membrane plane and 10 Å along the membrane normal.

Prior to the production phase, all RORγ systems underwent energy minimization using 3000 steps of steepest descent followed by 3000 steps of conjugate gradient minimization. The minimized systems were subsequently heated from 10 to 303 K over 500 ps under constant-volume conditions while applying positional restraints of 10 kcal mol^−1^ Å^−2^ to solute heavy atoms. Atomic velocities were randomly assigned according to a Maxwell–Boltzmann distribution at the initial temperature. Pressure equilibration was then performed for 500 ps under NPT conditions at 303 K and 1 bar while maintaining the same restraints. Positional restraints were gradually released through four additional 500 ps NPT stages, decreasing the restraint force constant by 2 kcal mol^−1^ Å^−2^ at each stage. The equilibrated systems were finally subjected to unrestrained 500 ns production simulations.

A similar protocol was adopted for the membrane-bound TGR5 systems, with modifications to accommodate the lipid environment. During equilibration, restraints applied to lipid atoms were removed immediately after the heating stage, whereas those applied to the protein and ligand were progressively reduced during four consecutive 4 ns NPT simulations. Subsequent production simulations were carried out for 500 ns in the NPT ensemble.

In all cases, the Langevin thermostat was used for temperature regulation throughout all simulations, while pressure control during NPT stages employed a Monte Carlo barostat. Long-range electrostatic interactions were treated using the particle mesh Ewald (PME) method, and a cutoff of 10 Å was applied for short-range nonbonded interactions.^66^ A timestep of 2 fs was employed during equilibration and increased to 4 fs for the production simulations through hydrogen mass repartitioning (HMR).^67^ Trajectory snapshots were saved every 60 ps. All simulations were performed using the pmemd.cuda module of Amber24.^66^

In addition, twelve independent 20 ns MD simulations were initiated for each system from representative structures extracted from the corresponding 500 ns production trajectories. In the TGR5 systems, interfacial buried water molecules relevant for ligand stabilization were introduced into the starting conformations used for topology and coordinate file generation by structural superposition from representative structures in which spontaneous water transfer into the binding pocket had been observed during the 500 ns simulations. The replicate 20 ns simulations followed the same protocol described above. Independent replicas were generated by assigning distinct initial velocity distributions during the heating stage, and the resulting trajectories were subsequently used for the MM-GBSA calculations.

#### 4.3.4 MM-GBSA free energy calculations

MM-GBSA free energy calculations were performed for the TGR5 and RORγ complexes to estimate ligand binding affinities and evaluate the contribution of conserved cavity water molecules to the observed SAR trends. The calculations were based on twelve independent 20 ns replicate MD simulations conducted for each system. Conventional MM-GBSA calculations were initially carried out after stripping all waters and ions from all trajectories, as well as lipid molecules from TGR5 trajectories. Additional Nwat-MMGBSA calculations were subsequently performed by retaining, in each frame, the single water molecule closest (*N* = 1) to the buried ligand polar atom identified during the corresponding 500 ns MD simulations as part of the receptor.^46^

Binding free energies were calculated with the GB-neck implicit solvent model using the *MMPBSA.py* module^68^ of AmberTools24,^60^ employing a salt concentration of 0.10 M and default internal (1.0) and external (78.5) dielectric constants. The single-trajectory protocol was adopted, in which the conformations of the isolated receptor and ligand were extracted from the corresponding complex trajectories. Configurational entropy contributions were neglected; therefore, the calculated binding energies are reported as effective free energies (Δ*G*_eff_).

#### 4.3.5 Trajectory analyses

The *cpptraj* module of AmberTools24 (ref. 60) was used to perform trajectory analyses. Root-mean-square deviations (RMSDs) were calculated using the RMSD command to evaluate the stability of the protein–ligand complexes over time. H-bond interactions were analysed with the H-bond command, applying geometric criteria of an acceptor–donor distance <3.5 Å and a donor–hydrogen–acceptor angle >120°.

Clustering analyses were performed using the agglomerative algorithm implemented in the cluster command to identify representative conformations from the replicate 500 ns MD trajectories. RMSD values of ligand heavy atoms and binding site residues were used as the similarity metric for clustering, and five clusters were generated for each protein–ligand system. The distance command was employed to compute interatomic distances throughout the trajectories, while the closest command was used to identify the water molecule nearest to the buried polar atoms of the ligands in each frame.

## Author contributions

Panagiota Stamou: data curation, formal analysis, investigation, methodology, supervision validation, visualization, writing – original draft, writing – review & editing; Alexander F. Perhal: data curation, formal analysis, investigation, methodology, supervision validation, visualization, writing – original draft, writing – review & editing; Patrik F. Schwarz: data curation, formal analysis, investigation, methodology, supervision validation, visualization, writing – original draft, writing – review & editing; Jorge Enrique Hernández González: data curation, formal analysis, investigation, visualization, funding acquisition, writing – review & editing; Justin Weal: data curation, formal analysis, investigation, writing – review & editing; Ya Chen: data curation, formal analysis, investigation, writing – review & editing; Alexios Leandros Skaltsounis: conceptualization, writing – review & editing; Maria Halabalaki: conceptualization, supervision, writing – review & editing; Johannes Kirchmair, Verena M. Dirsch: conceptualization, project administration, funding acquisition, resources, supervision, writing – review & editing; Ioannis K. Kostakis: conceptualization, project administration, supervision, writing – review & editing.

## Conflicts of interest

The authors declare that they have no known competing financial interests or personal relationships that could have appeared to influence the work reported in this paper.

## Supplementary Material

MD-OLF-D6MD00471G-s001

## Data Availability

The data supporting this article have been included as part of the supplementary information (SI). Supplementary information: MD simulation and MM-GBSA binding data for compounds 1, 5, 7 and 8, a cell-viability assay, and full NMR characterization of the synthesized derivatives. See DOI: https://doi.org/10.1039/d6md00471g.
