# Supplementary material for: Oleanolic acid-derived triterpenoids with dual TGR5 agonistic and RORγ inverse agonistic activity
Source: RSC Med Chem. 2026 Oct 6. Online ahead of print. doi: 10.1039/d6md00471g (PMC13670034; doi:10.1039/d6md00471g)
Supplement: Supplementary file 1 [file MD-OLF-D6MD00471G-s001.pdf]

Supplementary Information (SI) for RSC Medicinal Chemistry.
This journal is © The Royal Society of Chemistry

Supplementary materials

Oleanolic acid-derived triterpenoids with dual TGR5 agonistic and RORγ inverse agonistic activity

Panagiota Stamou[a], Alexander F. Perhal[b], Patrik F. Schwarz[b], Jorge Enrique Hernández González[b,c], Justin Weal[b], Ya Chen[b], Alexios Leandros Skaltsounis[d], Maria Halabalaki[d], Johannes Kirchmair[b], Verena M. Dirsch[b], Ioannis K. Kostakis[a.]

[a]Department of Pharmacy, Division of Pharmaceutical Chemistry, National and Kapodistrian University of Athens, Panepistimiopolis, Zografou, 15771, Athens, Greece
[b]Department of Pharmaceutical Sciences, University of Vienna, 1090 Vienna, Austria
[c]Multiuser Center for Biomolecular Innovation, São Paulo State University - UNESP, São José do Rio Preto, SP, Brazil
[d]Department of Pharmacy, Division of Pharmacognosy and Natural Products Chemistry, National and Kapodistrian University of Athens, Panepistimiopolis Zografou, Athens, 15771, Greece

Contents

Table S1. H-bond occupancies at the interfaces of TGR5 complexes during the 500 ns MD simulations 3

Table S2. Water bridge occupancies at the interfaces of TGR5 complexes during the 500 ns MD simulations........ 3

Table S3. MM-GBSA free energy calculations TGR5 in complex with the analyzed compounds 4

Table S4. H-bond occupancies at the interfaces of RORγ complexes during the 500 ns MD simulations 4

Table S5. Water bridge occupancies at the interfaces of RORγ complexes during the 500 ns MD simulations........ 4

Table S6. MM-GBSA free energy calculations RORγ in complex with the analyzed compounds 5

Figure S1: Resazurin conversion assay of the pentacyclic triterpenoids 1, 7, and 8. 6

Figure S2. Structural representation of the selected docking poses of compounds bound to the TGR5 crystal structure 7CFN........ 6

Figure S3. RMSD of ligands in TGR5 complexes during 500 ns MD simulations 7

Figure S4. Distance distributions between buried polar atoms of the analyzed compounds, residue Y240, and interfacial water molecules in TGR5 complexes 7

Figure S5. Structural representation of the selected docking poses of compounds bound to the RORγ crystal structure 6J3N 8

Figure S6. RMSD of ligands in RORγ complexes during 500 ns MD simulations 8

Figure S7. Distance distributions between buried polar atoms of the analyzed compounds, residue H479, and interfacial water molecules in RORγ complexes 9

Figure S8: *^{1}H (600 MHz, $CDCl_3$) spectrum of 1.* 9

Figure S9: ^{1}H (400 MHz, $CDCl_3$) spectrum of 4. 10

Figure S10: ^{1}H (600 MHz, $CDCl_3$) spectrum of 5. 10

Figure S11: ^{1}H (400 MHz, $CDCl_3$) spectrum of 6. 11

Figure S12: ^{1}H (600 MHz, $CDCl_3$) spectrum of 7. 11

Figure S*13*: ^{1}H (400 MHz, $CDCl_3$) spectrum of 8. 12

Figure S*14*: ^{13}*C* (100 MHz, $CDCl_3$) spectrum of 8. 13

Figure S*15*: COSY (400 MHz, $CDCl_3$) spectrum of 8........ 14

Figure S*16*: HSQC (400 MHz, $CDCl_3$) spectrum of 8........ 15

Figure S*17*: HMBC (400 MHz, $CDCl_3$) spectrum of 8. 16

Figure S18: ^{1}H (600 MHz, $CDCl_3$) spectrum of 9. 17

Figure S1*9*: ^{13}C (150 MHz, $CDCl_3$) spectrum of 9. 18

Figure S*20*: COSY (600 MHz, $CDCl_3$) spectrum of 9........ 19

Figure S*21*: HSQC (600 MHz, $CDCl_3$) spectrum of 9........ 20

Figure S*22*: HMBC (600 MHz, $CDCl_3$) spectrum of 9. 21

Table S1. H-bond occupancies at the interfaces of TGR5 complexes during the 500 ns MD simulations[a]

Compound	Protein Donor	Ligand Acceptor	Occupancy (%)
1	Y89(OH)	O1	68.84
	S157(OG)	O3	62.51
	N93(OD1)	O2	56.74
	Y89(OH)	O3	39.99
	S157(OG)	O1	38.34
5	S157(OG)	O3	95.72
	Y89(OH)	O1	85.20
7	S157(OG)	O3	98.92
	Y89(OH)	O1	95.32
	N93(ND2)	O2	45.70
	Y240(OH)	O2	26.32
8	Y89(OH)	O1	76.74
	Y89(OH)	O2	71.99
	S157(OG)	O2	54.84
	S157(OG)	O1	41.20
	Y240(OH)	N1	29.59

[a]Only H-bonds with occupancy ≥20% are reported

Table S2. Water bridge occupancies at the interfaces of TGR5 complexes during the 500 ns MD simulations[a]

Compound	Water bridge residues	Lingand polar atom	Occupancy (%)[b]
1	L74(O)	O1/2 (carboxyl)	37.44
	S157(OG)	O1/2(carboxyl)	29.06
	Q158(NE2)–A250(O)[c]	O1/2(carboxyl)	22.30
	S157(OG)–Q158(OE1)	O1/2 (carboxyl)	21.61
5	E169(OE1)–Y240(OH)	O3 (carbonyl)	38.45
	S157(OG)	O1/2 (carboxyl)	34.08
	L74(O)	O1/2 (carboxyl)	30.36
	Q158(NE2)–A250(O)	O1/2 (carboxyl)	27.49
	Y240(OH)	O3(carbonyl)	23.06
7	L74(O)	O1/2 (carboxyl)	54.01
	S157(OG)–Q158(OE1)	O1/2 (carboxyl)	47.02
	Q158(NE2)–A250(O)	O1/2 (carboxyl)	40.30
	S157(OG)	O1/2 (carboxyl)	30.63
	Q158(NE2)	O1/2 (carboxyl)	30.59
8	L74(O)	O1/2 (carboxyl)	75.02
	Q158(NE2)	O1/2 (carboxyl)	41.64
	S157(OG)	O1/2 (carboxyl)	29.10

[a]Only water bridges with occupancy ≥20% are reported. [b]Water bridge occupancy is expressed as the percentage of trajectory frames in which the water bridge was observed. [c]When two residues are listed, the water bridge involves a single water molecule simultaneously interacting with both protein residues and the ligand polar atom.

Table S3. MM-GBSA free energy calculations TGR5 in complex with the analyzed compounds[a]

Cpnd	ΔE_{vdW}[b]	ΔE_{el}[c]	ΔE_{GB}[d]	ΔE_{surf}[e]	ΔG_{eff}[f]	$\Delta\Delta G$[g]
1	-56.97 (0.16)[h]	-174.50 (0.61)	175.15 (0.52)	-7.11 (0.01)	-63.43 (0.22)	-
5	-55.87 (0.12)	-173.06 (0.81)	174.69 (0.85)	-7.05 (0.01)	-61.30 (0.25)	2.13 (0.33)
7	-57.10 (0.16)	-169.45 (1.20)	171.06 (1.00)	-7.35 (0.02)	-62.90 (0.32)	0.53 (0.39)
8	-56.29 (0.29)	-176.48 (0.80)	175.94 (0.79)	-7.50 (0.03)	-64.33 (0.28)	-0.90 (0.35)

[a]All values are expressed in kcal/mol. [b]van der Waals contribution. [c]Electrostatic contribution. [d]Polar solvation free energy calculated using the generalized Born model GB-neck. [e]Nonpolar solvation contribution estimated from the solvent-accessible surface area. [f]Effective binding free energy, calculated as the sum of the previous energy components. [g]Relative binding free energy with respect to **1**. [h]Values in parentheses correspond to the SEM.

Table S4. H-bond occupancies at the interfaces of RORγ complexes during the 500 ns MD simulations[a]

Compound	Protein Donor	Ligand Acceptor	Occupancy (%)
1	H323(ND1)	O1 (carboxyl)	77.22
	H323(ND1)	O2 (carboxyl)	61.27
5	H323(ND1)	O1 (carboxyl)	80.87
	H323(ND1)	O2 (carboxyl)	75.03
7	H323(ND1)	O1 (carboxyl)	83.14
	H323(ND1)	O2 (carboxyl)	71.96
	H479(ND1)	O3 (hydroxyl)	53.22
8	H323(ND1)	O1 (carboxyl)	66.96
	H323(ND1)	O2 (carboxyl)	65.15
	H479(ND1)	N1 (cyano)	61.78

[a]Only H-bonds with occupancy ≥20% are reported

Table S5. Water bridge occupancies at the interfaces of RORγ complexes during the 500 ns MD simulations[a]

Compound	Water bridge residues	Lingand polar atom	Occupancy (%)[b]
1	E379(OE1/2)	O1/2 (carboxyl)	78.24
	Q286(OE1)–H323(ND1/NE2)[c]	O1/2(carboxyl)	72.41
	H479(ND1/NE2)	O3 (hydroxyl)	69.42
5	E379(OE1/2)	O1/2 (carboxyl)	59.09
	H479(ND1/NE2)	O3 (carbonyl)	56.14
	H323(ND1/NE2)	O1/2 (carboxyl)	54.51
	Q286(–H323(ND1/NE2)	O1/2 (carboxyl)	29.11
	F377(O)	O1/2 (carboxyl)	21.20
7	H323(ND1/NE2)	O1/2 (carboxyl)	83.93
	E379(OE1/OE2)	O1/2 (carboxyl)	76.92
	F377(O)	O1/2 (carboxyl)	45.04
8	E379(OE1;OE2)	O1/2 (carboxyl)	63.57
	Q286(OE1)–H323(ND1/NE2)	O1/2 (carboxyl)	63.33
	F377(O)	O1/2 (carboxyl)	22.90
	H323(ND1/NE2)	O1/2 (carboxyl)	21.85

[a]Only water bridges with occupancy ≥20% are reported. [b]Water bridge occupancy is expressed as the percentage of trajectory frames in which the water bridge was observed. [c]When two residues are listed, the water bridge involves a single water molecule simultaneously interacting with both protein residues and the ligand polar atom.

Table S6. MM-GBSA free energy calculations RORγ in complex with the analyzed compounds[a]

Cpnd	ΔE_{vdW}[b]	ΔE_{el}[c]	ΔE_{GB}[d]	ΔE_{surf}[e]	ΔG_{eff}[f]	$\Delta\Delta G$[g]
1	-58.10 (0.18)[h]	-91.37 (1.26)	104.78 (1.07)	-7.24 (0.02)	-51.93 (0.28)	-
5	-56.24 (0.40)	-80.79 (4.19)	94.28 (3.82)	-6.76 (0.07)	-49.50 (0.54)	2.43 (0.61)
7	-56.78 (0.09)	-88.95 (1.97)	102.49 (1.73)	-6.86 (0.02)	-50.11 (0.32)	1.82 (0.42)
8	-58.35 (0.17)	-94.94 (0.97)	107.62 (0.88)	-7.47 (0.01)	-53.14 (0.28)	-1.21 (0.39)

[a]All values are expressed in kcal/mol. [b]van der Waals contribution. [c]Electrostatic contribution. [d]Polar solvation free energy calculated using the generalized Born model GB-neck. [e]Nonpolar solvation contribution estimated from the solvent-accessible surface area. [f]Effective binding free energy, calculated as the sum of the previous energy components. . [g]Relative binding free energy with respect to **1**. [h]Values in parentheses correspond to the SEM.

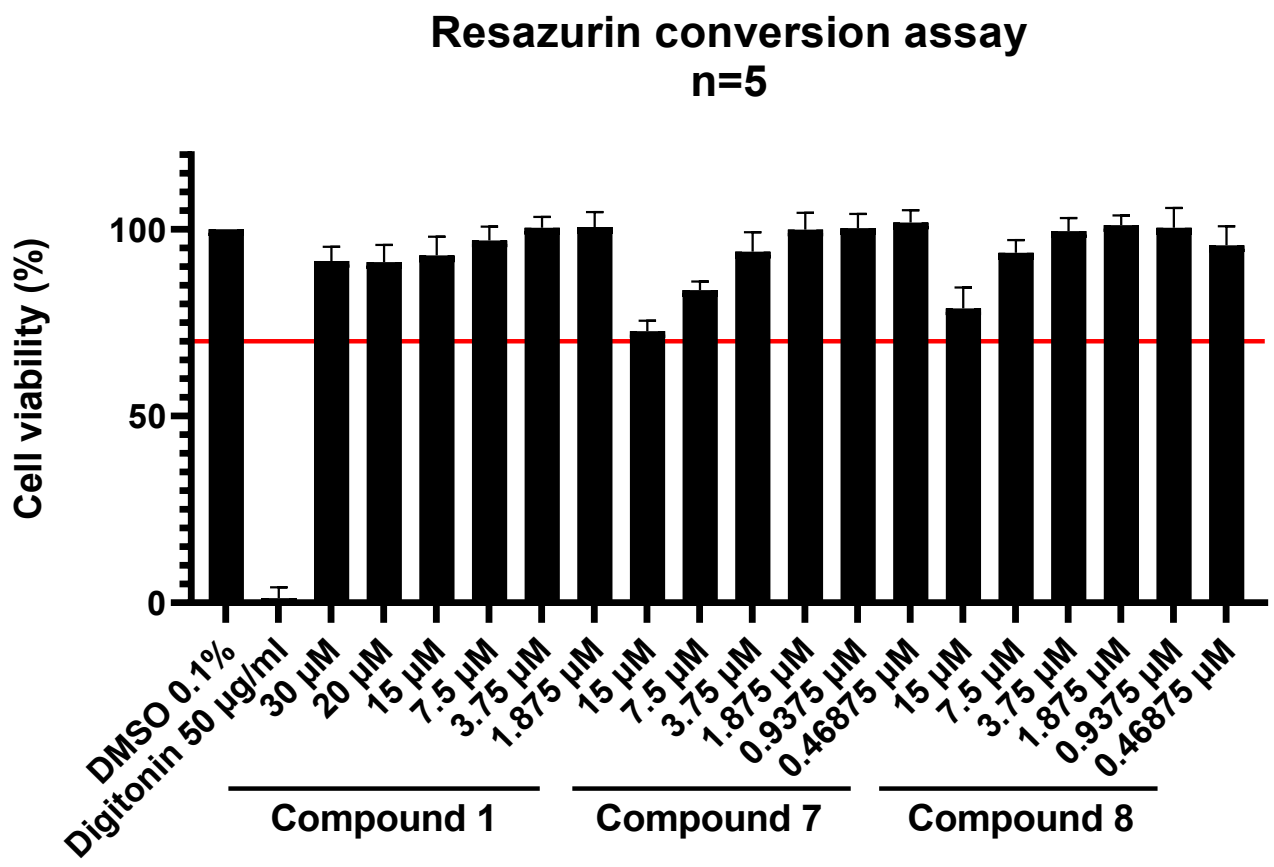

Figure S1: Resazurin conversion assay of the pentacyclic triterpenoids 1, 7, and 8. Cells were treated with the indicated compounds or the cytotoxic positive control digitonin (50 µg/mL) for 18 h before addition of resazurin (10 µg/mL) for 5 h. Fluorescence was measured at an excitation wavelength of 535 nm and an emission wavelength of 590 nm. Data are shown as mean ± SD from five independent biological replicates (n = 5), each measured in technical quadruplicate. The cytotoxicity threshold was set at 70% (red line) according to the ISO 10993-5 guideline.

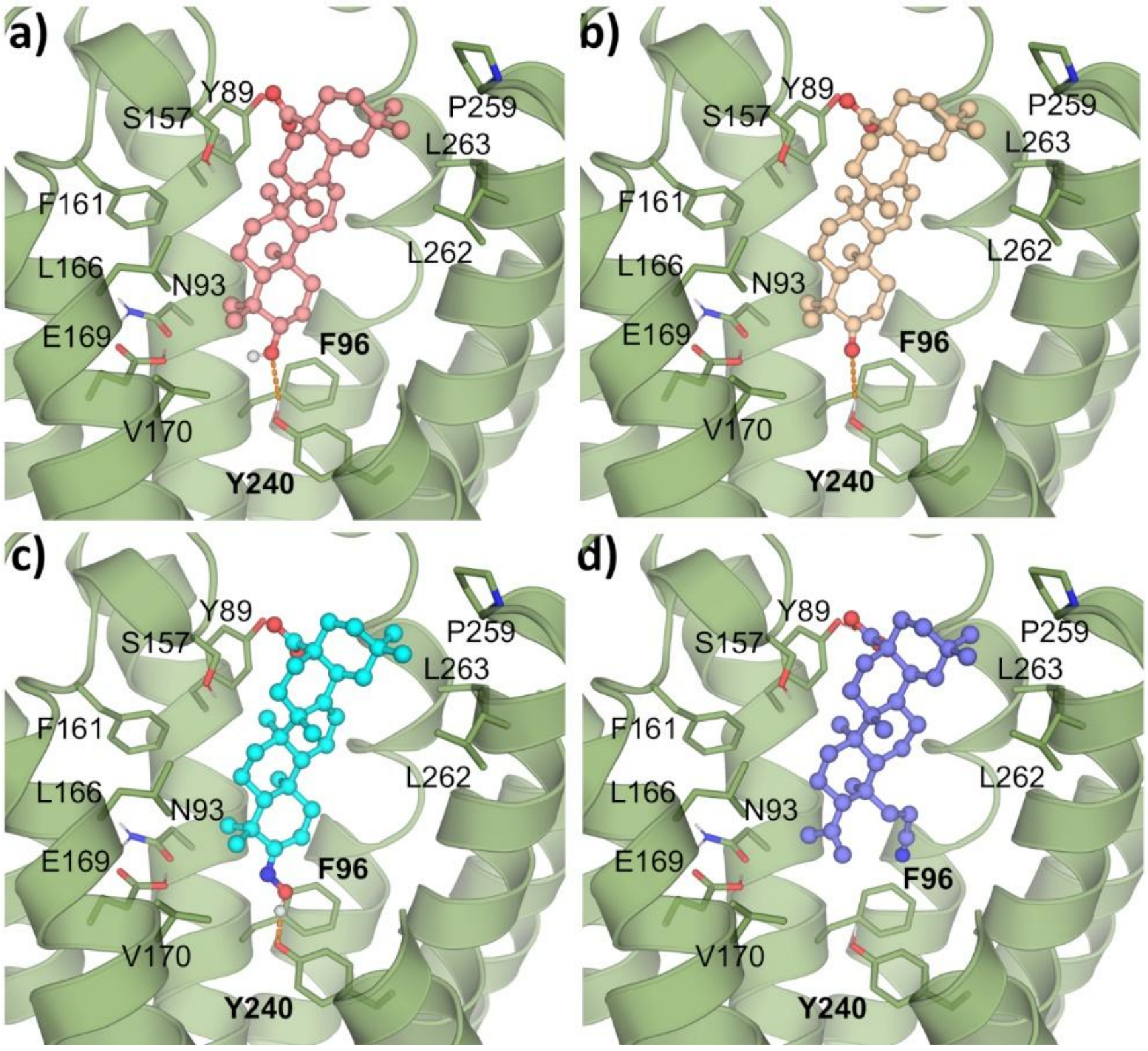

Figure S2. Structural representation of the selected docking poses of compounds bound to the TGR5 crystal structure 7CFN: **a) 1**; **b) 5**; **c) 7**; and **d) 8**. Ligands are shown in ball-and-stick representation, while key active-site residues are displayed as sticks and labeled accordingly. Residues F96 and Y240, which participate in stacking interactions, are highlighted in bold. H-bond interactions are depicted as orange dashed lines.

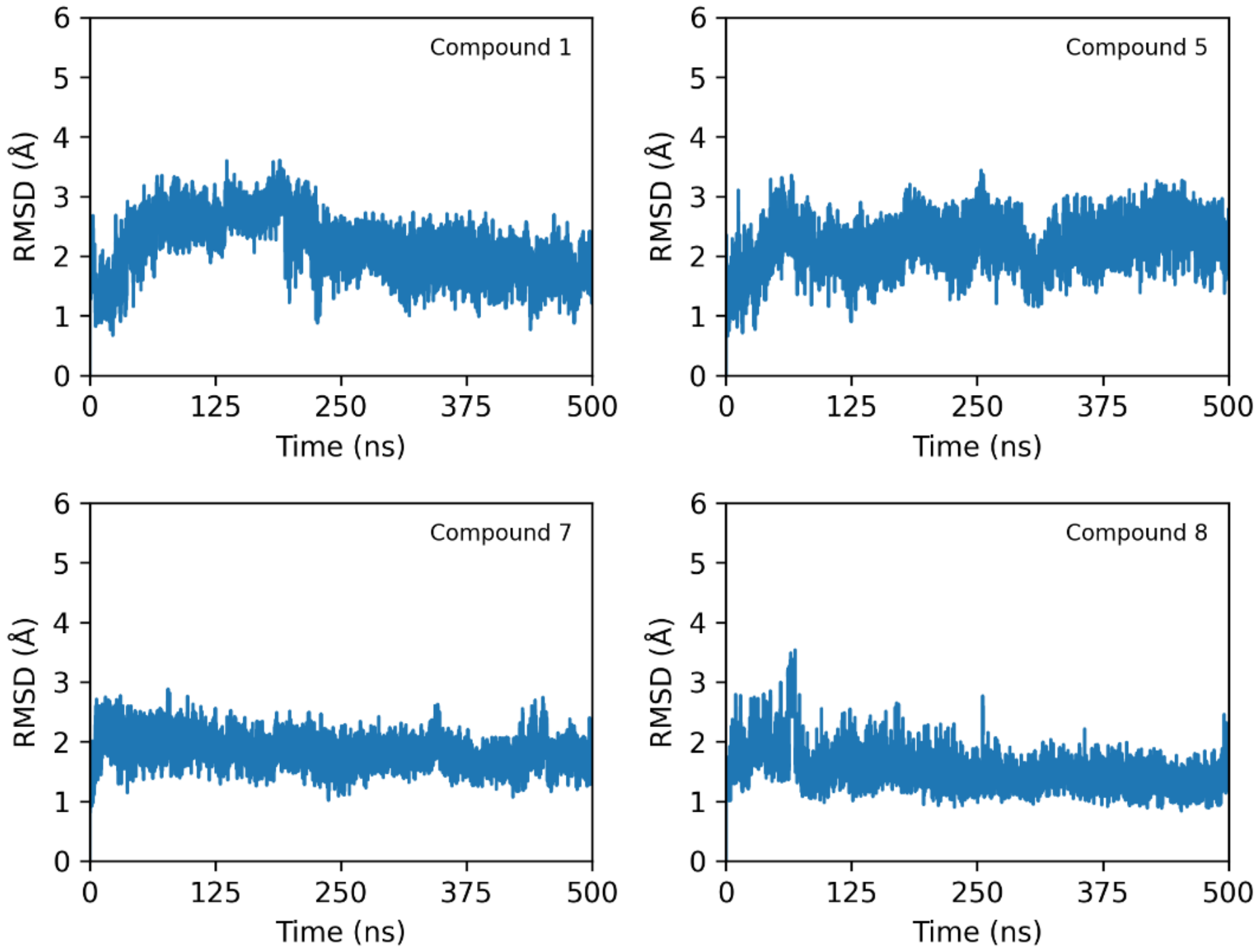

Figure S3. RMSD of ligands in TGR5 complexes during 500 ns MD simulations. RMSD values were calculated for the heavy atoms of each ligand relative to its docking pose, after fitting the protein backbone atoms of all trajectories to the initial structure.

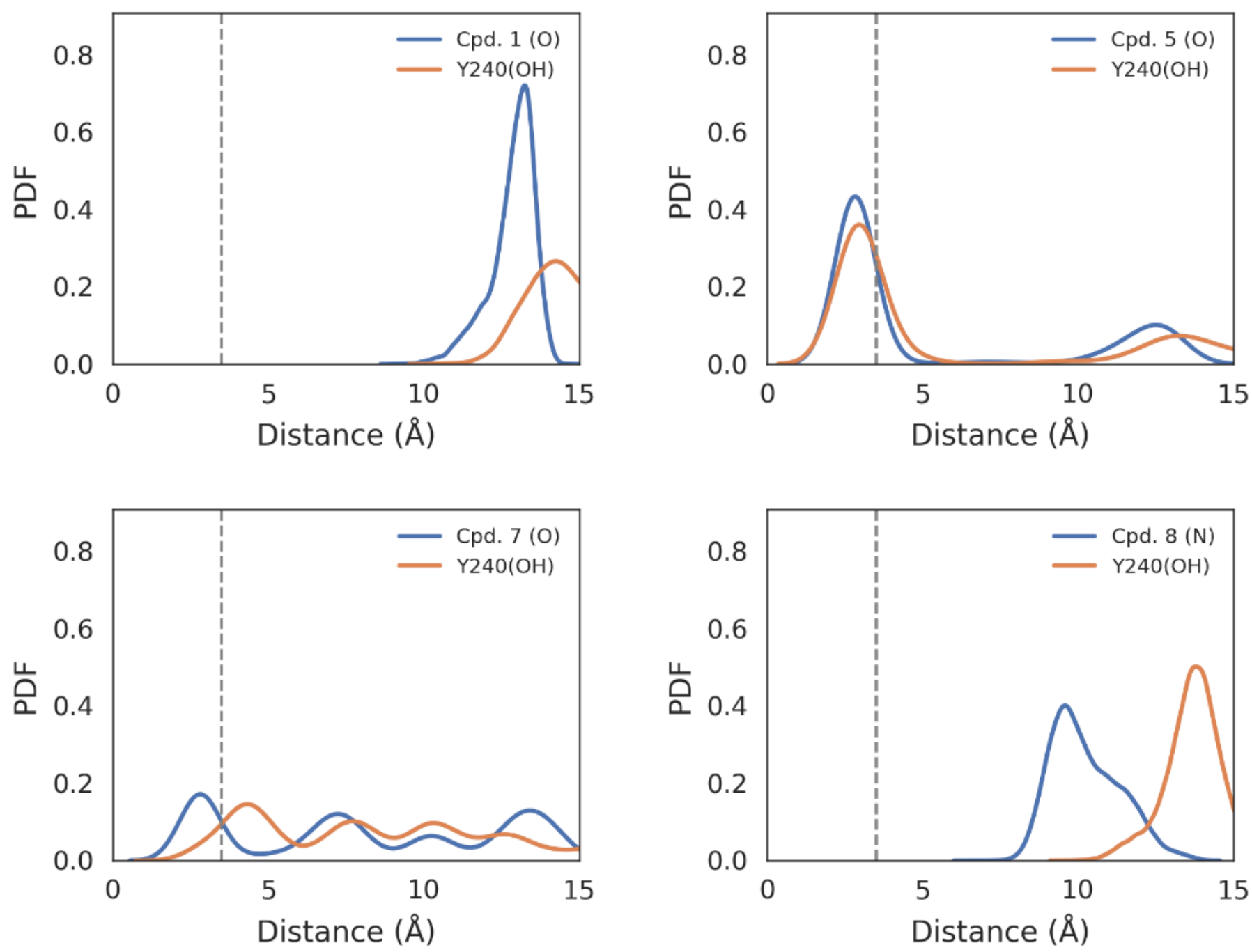

Figure S4. Distance distributions between buried polar atoms of the analyzed compounds, residue Y240, and interfacial water molecules in TGR5 complexes. The distributions depicted in blue represent the distances between the buried polar atoms of the compounds (hydroxyl O in **1** and **7**; carbonyl O in **2**; and cyano N in **8**) and the O atom of the nearest water molecule during the MD simulations. The distributions in orange represent the distances between the hydroxyl O atom of Y240 and the O atom of the water molecule closest to the ligands' buried polar atoms during the MD simulations. The vertical dashed lines in each graph indicate the cutoff distance for H-bond formation (3.5 Å)

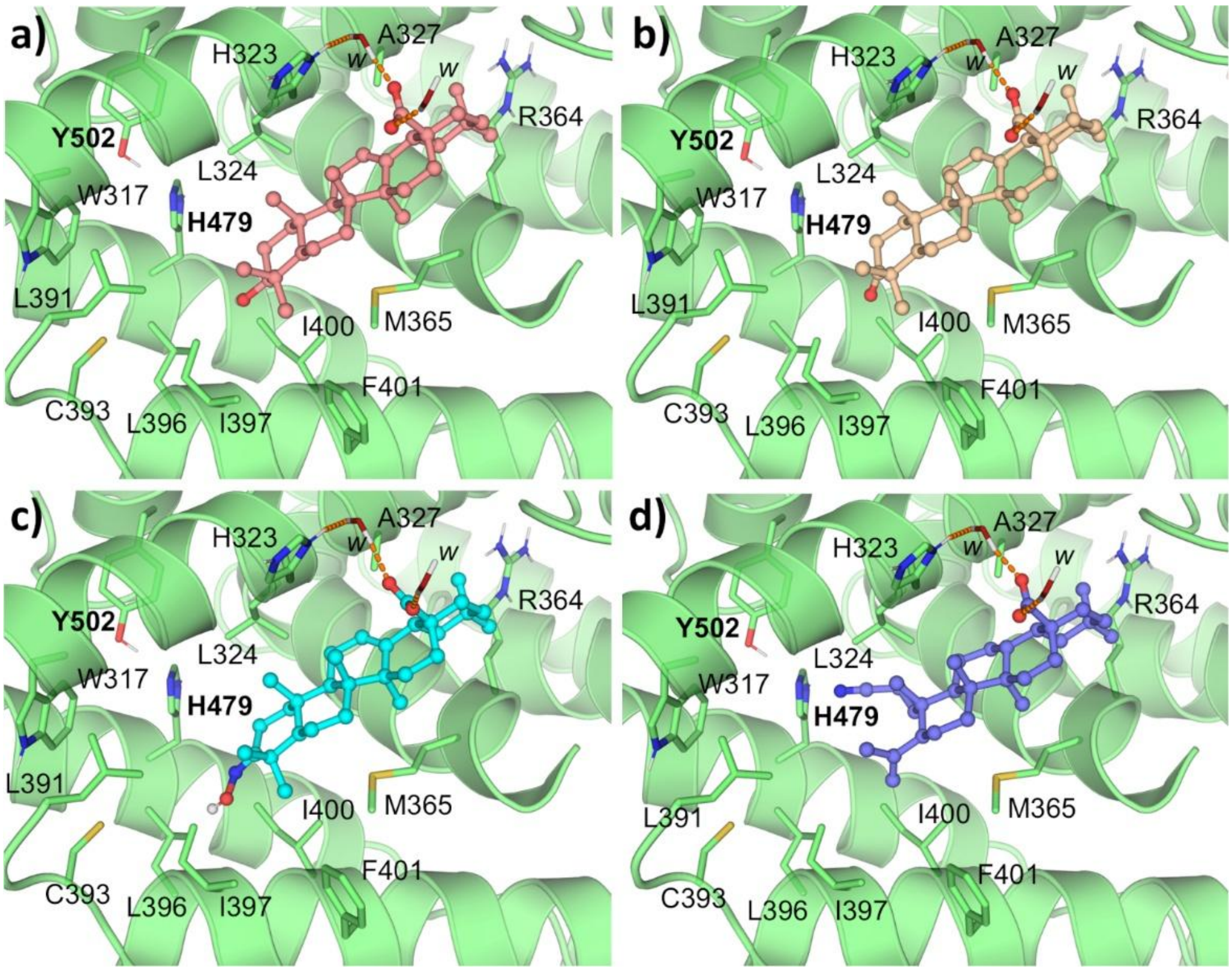

Figure S5. Structural representation of the selected docking poses of compounds bound to the RORγ crystal structure 6J3N: **a) 1**; **b) 5**; **c) 7**; and **d) 8**. Ligands are shown in ball-and-stick representation, while key active-site residues are displayed as sticks and labeled accordingly. Residues Y502 and H479, which form the agonist lock, are highlighted in bold. Crystallographic water molecules retained as part of the receptor during docking are shown as sticks and labeled (*w*) accordingly. H-bond interactions are depicted as orange dashed lines.

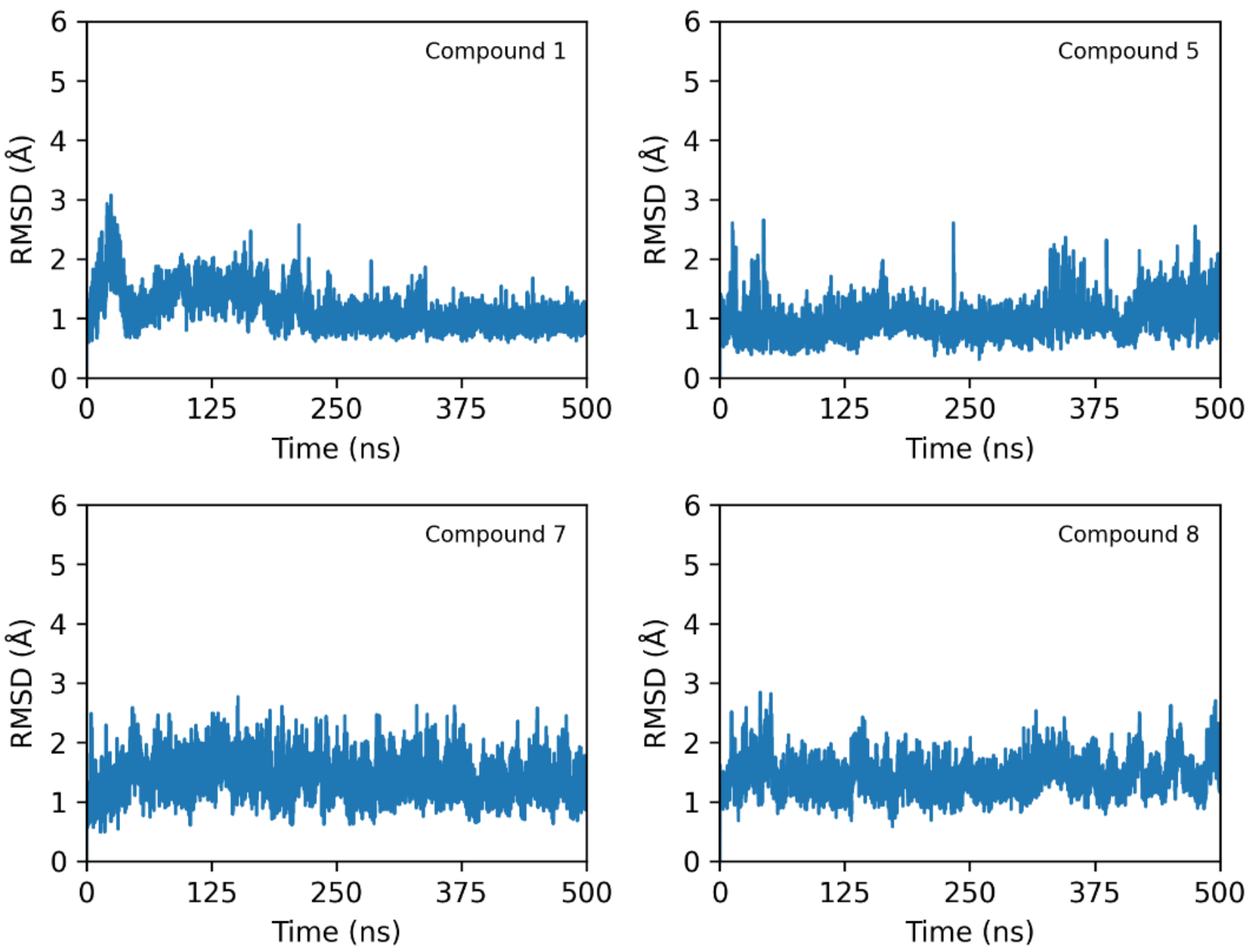

Figure S6. RMSD of ligands in RORγ complexes during 500 ns MD simulations. RMSD values were calculated for the heavy atoms of each ligand relative to its docking pose, after fitting the protein backbone atoms of all trajectories to the initial structure.

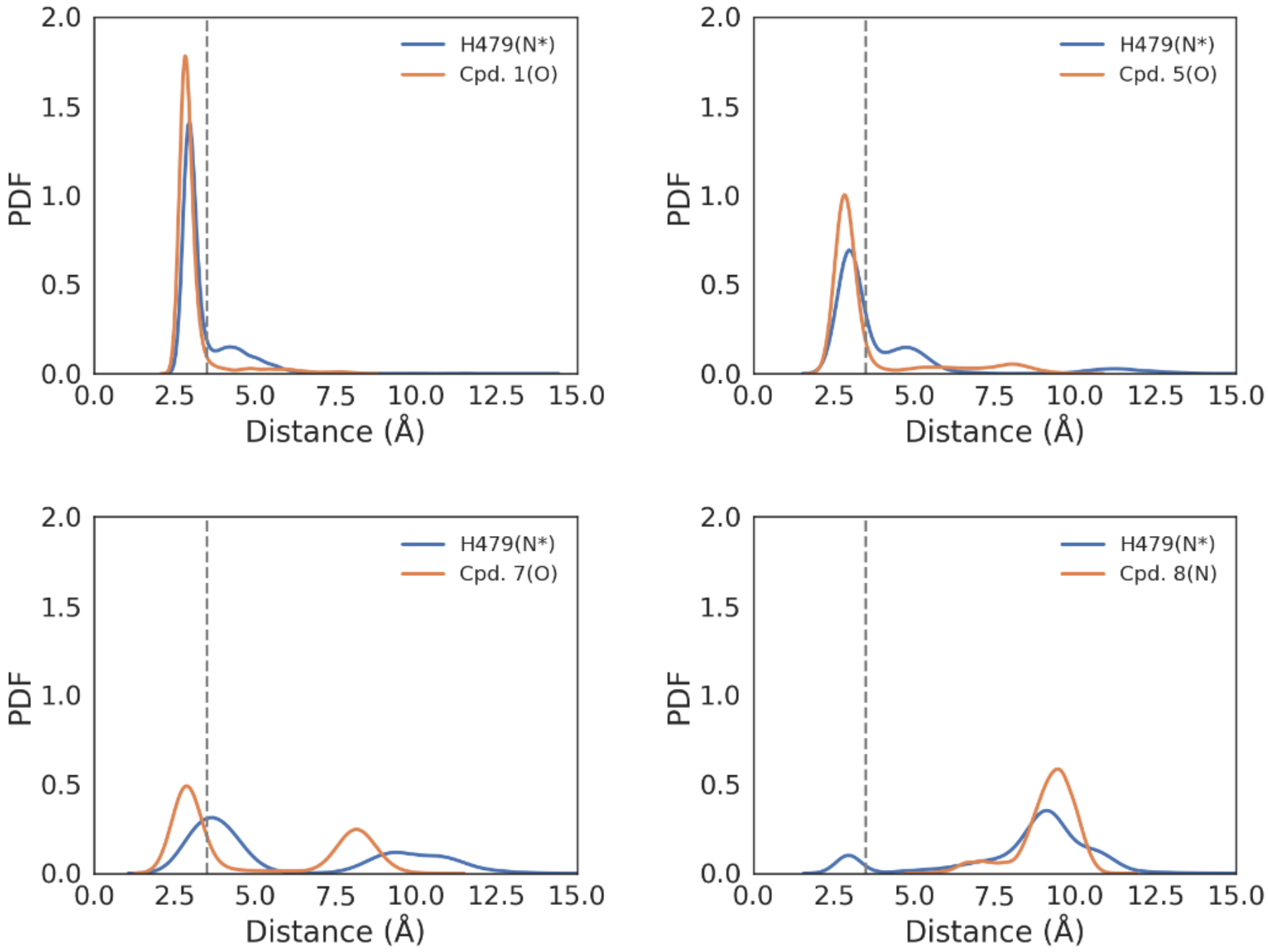

Figure S7. Distance distributions between buried polar atoms of the analyzed compounds, residue H479, and interfacial water molecules in RORγ complexes. The distributions depicted in blue represent the distances between the buried polar atoms of the compounds (hydroxyl O in **1** and **7**; carbonyl O in **2**; and cyano N in **8**) and the O atom of the nearest water molecule during the MD simulations. The distributions in orange represent the minimum distances between either imidazole N atom of H479 (ND1 or NE2) and the O atom of the water molecule closest to the ligands' buried polar atoms during the MD simulations. The vertical dashed lines in each graph indicate the cutoff distance for H-bond formation (3.5 Å)

Oleanolic acid (**1**)

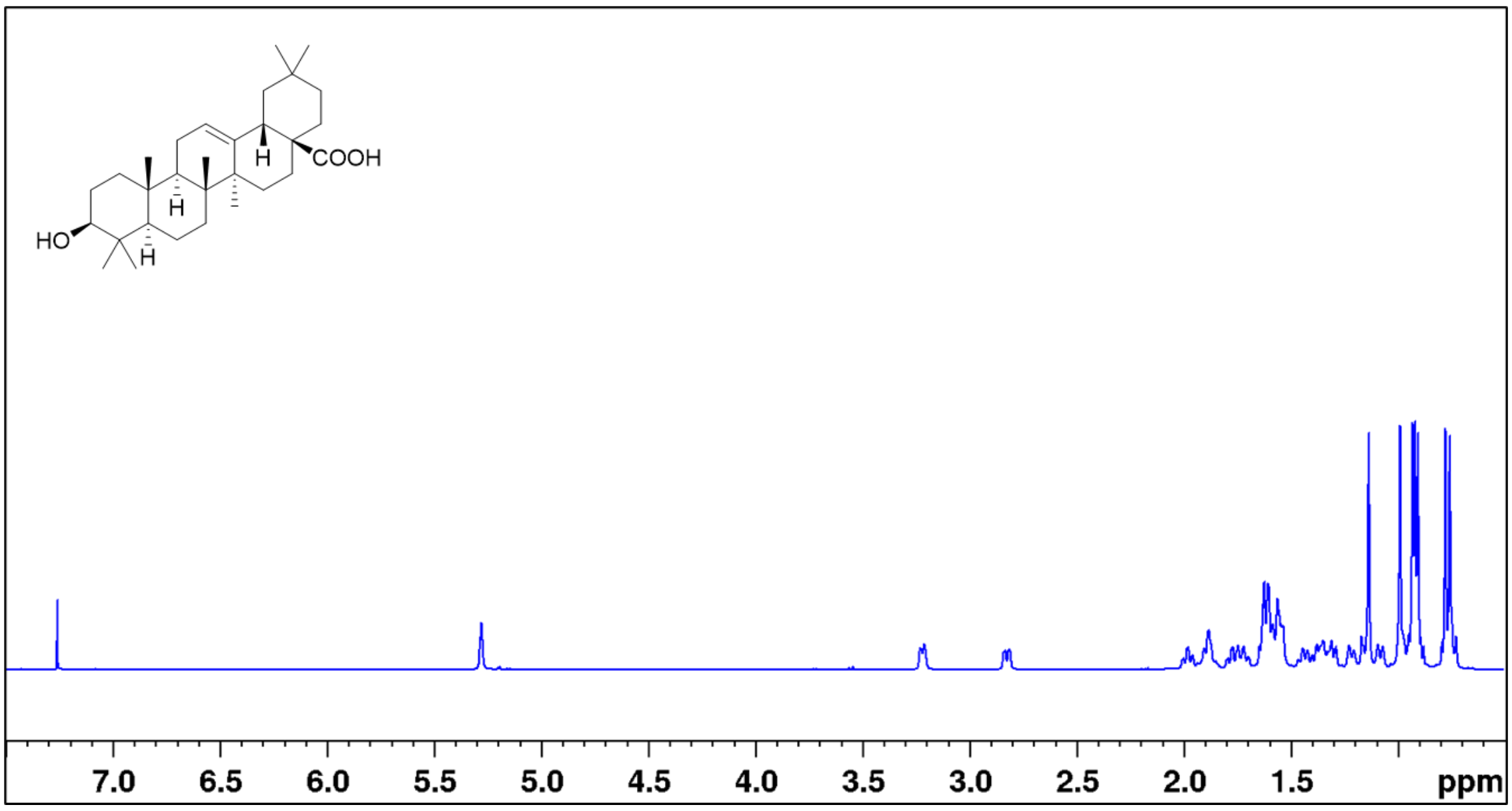

Figure S8: ***^{1}H (600 MHz, $CDCl_3$) spectrum of 1.***

δ-Oleanolic acid (**4**)

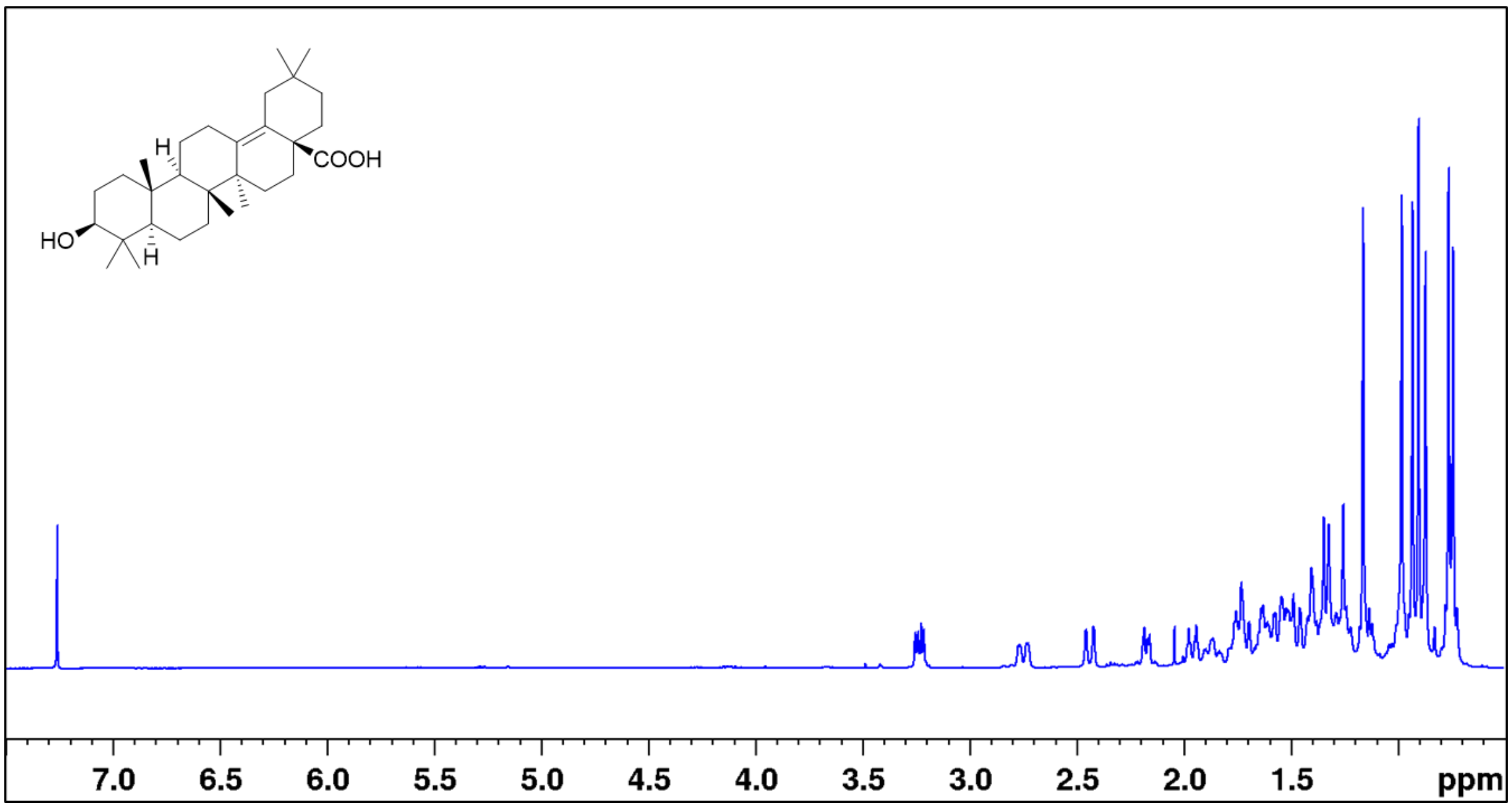

Figure S9: ^{1}H (400 MHz, $CDCl_3$) spectrum of **4**.

Oleanonic acid (**5**)

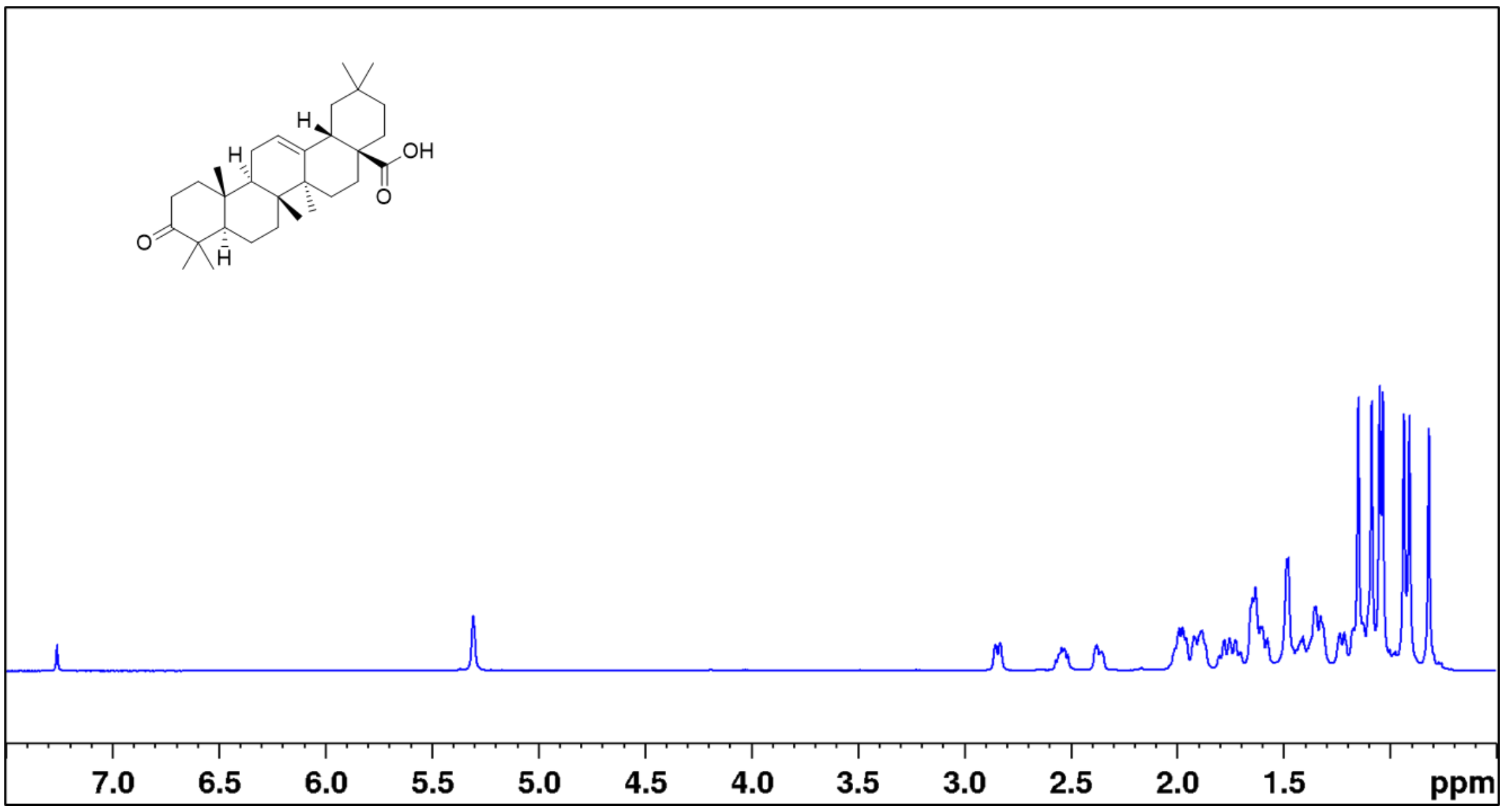

Figure S10: ^{1}H (600 MHz, $CDCl_3$) spectrum of **5**.

2-Oxoolean-12-en-28-amide (**6**)

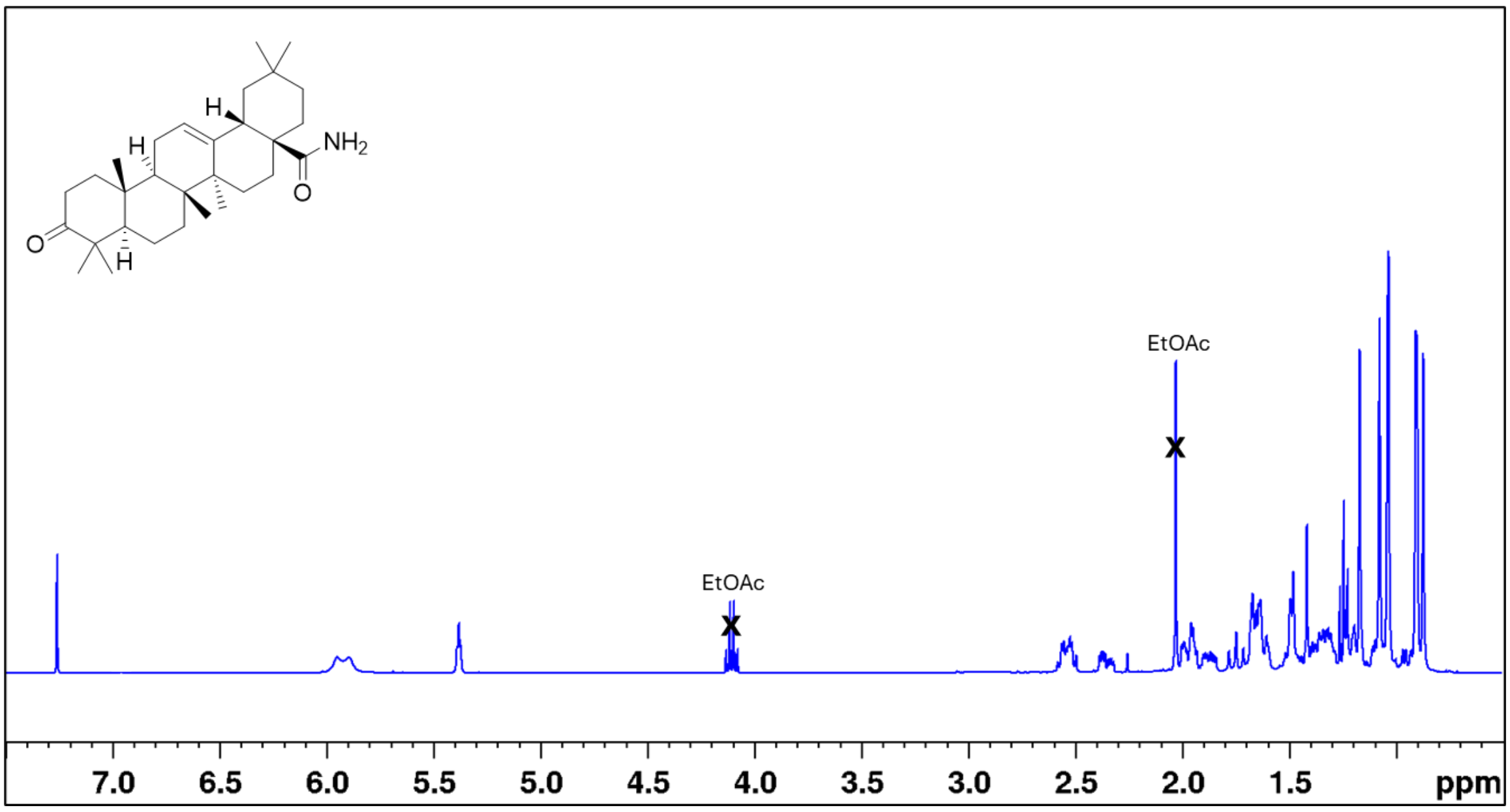

Figure S11: ^{1}H (400 MHz, $CDCl_3$) spectrum of **6**.

3-Hydroxyimino-olean-12-en-28-oic acid (**7**)

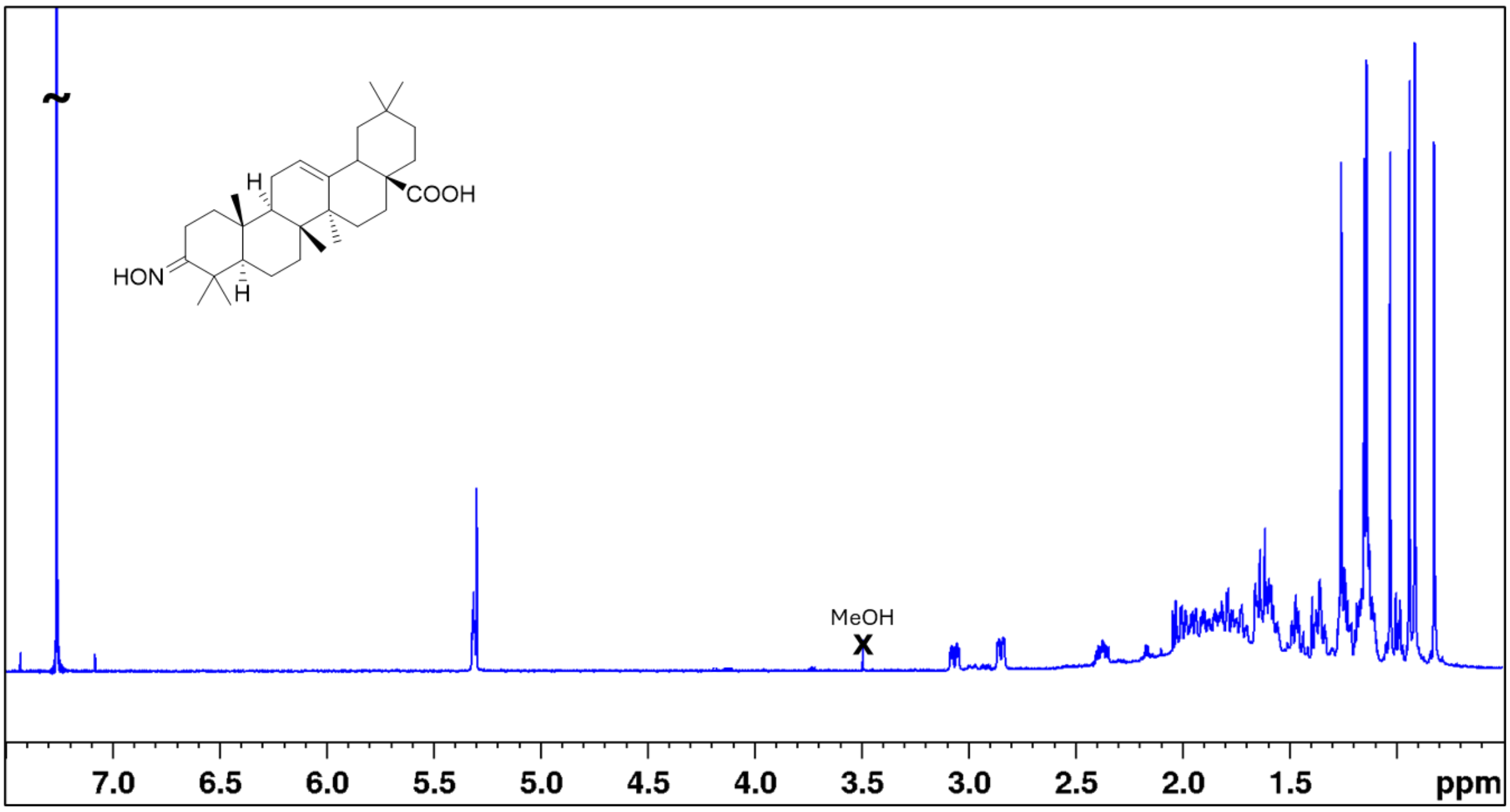

Figure S12: ^{1}H (600 MHz, $CDCl_3$) spectrum of 7.

3-Cyano-3,4-seco-4-ylidenolean-12-en-28-oic acid (**8**)

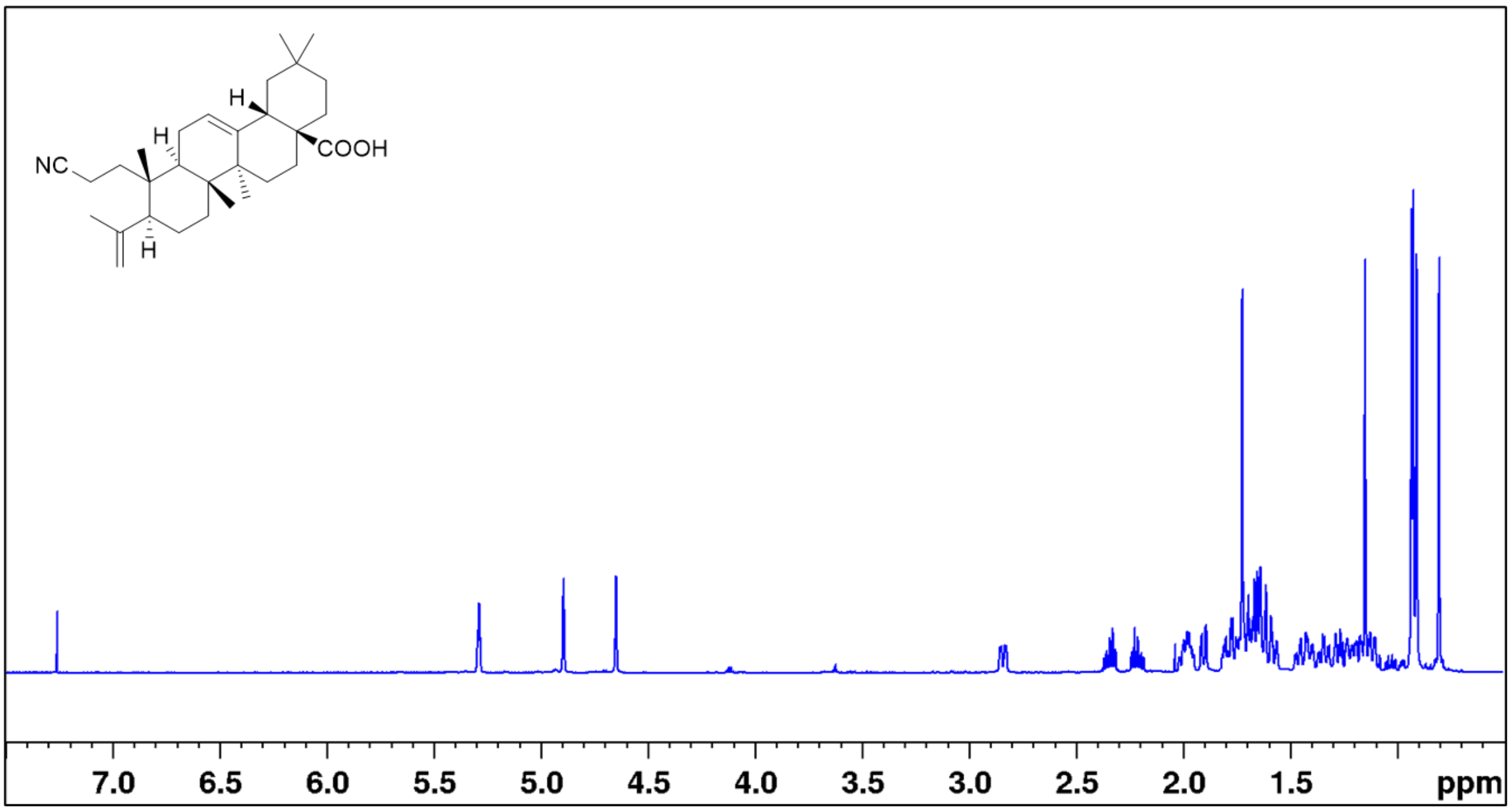

Figure S*13*: ^{1}H (400 MHz, $CDCl_3$) spectrum of **8**.

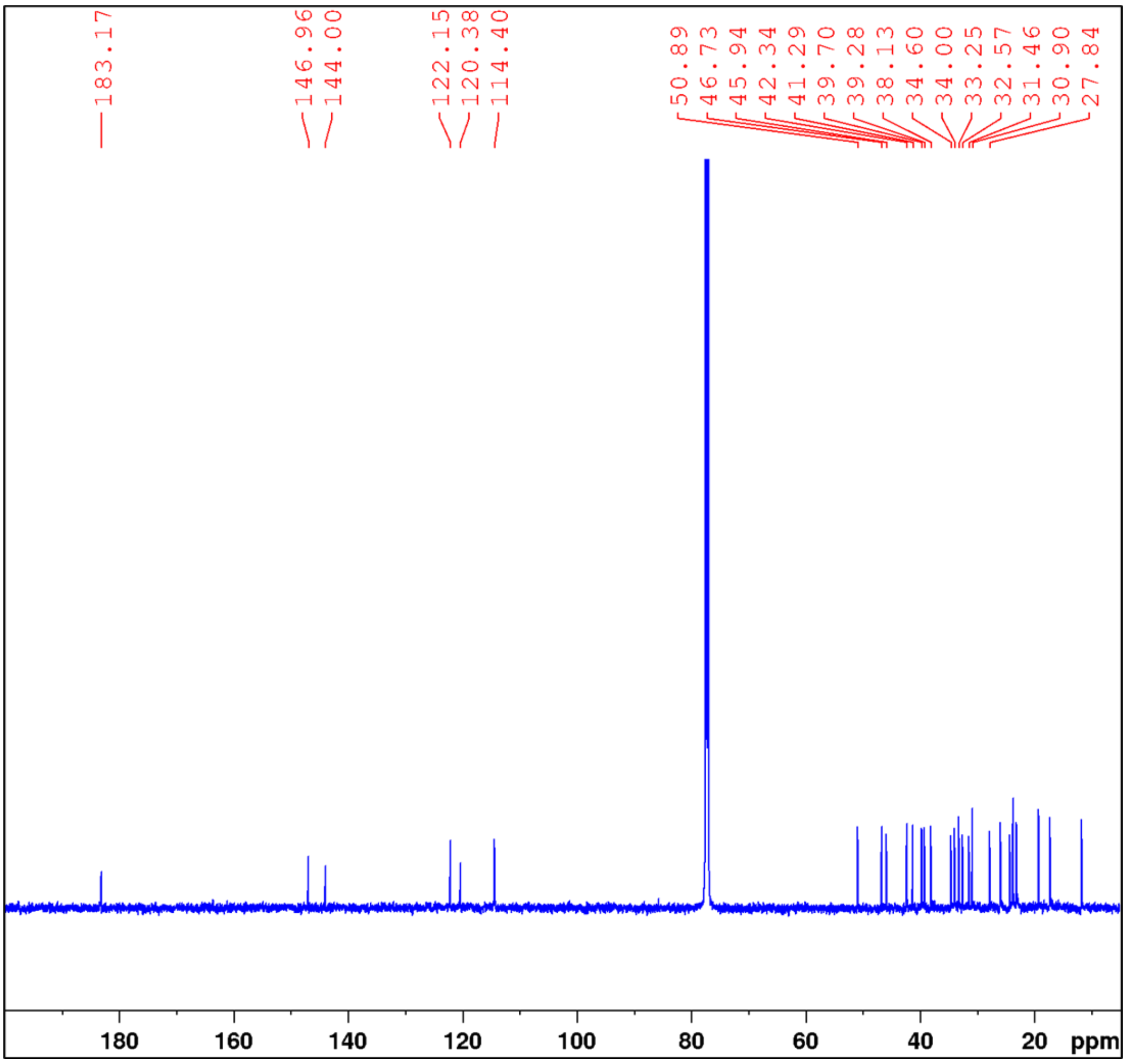

Figure S*14*: ^{13}C (100 MHz, $CDCl_3$) spectrum of **8**.

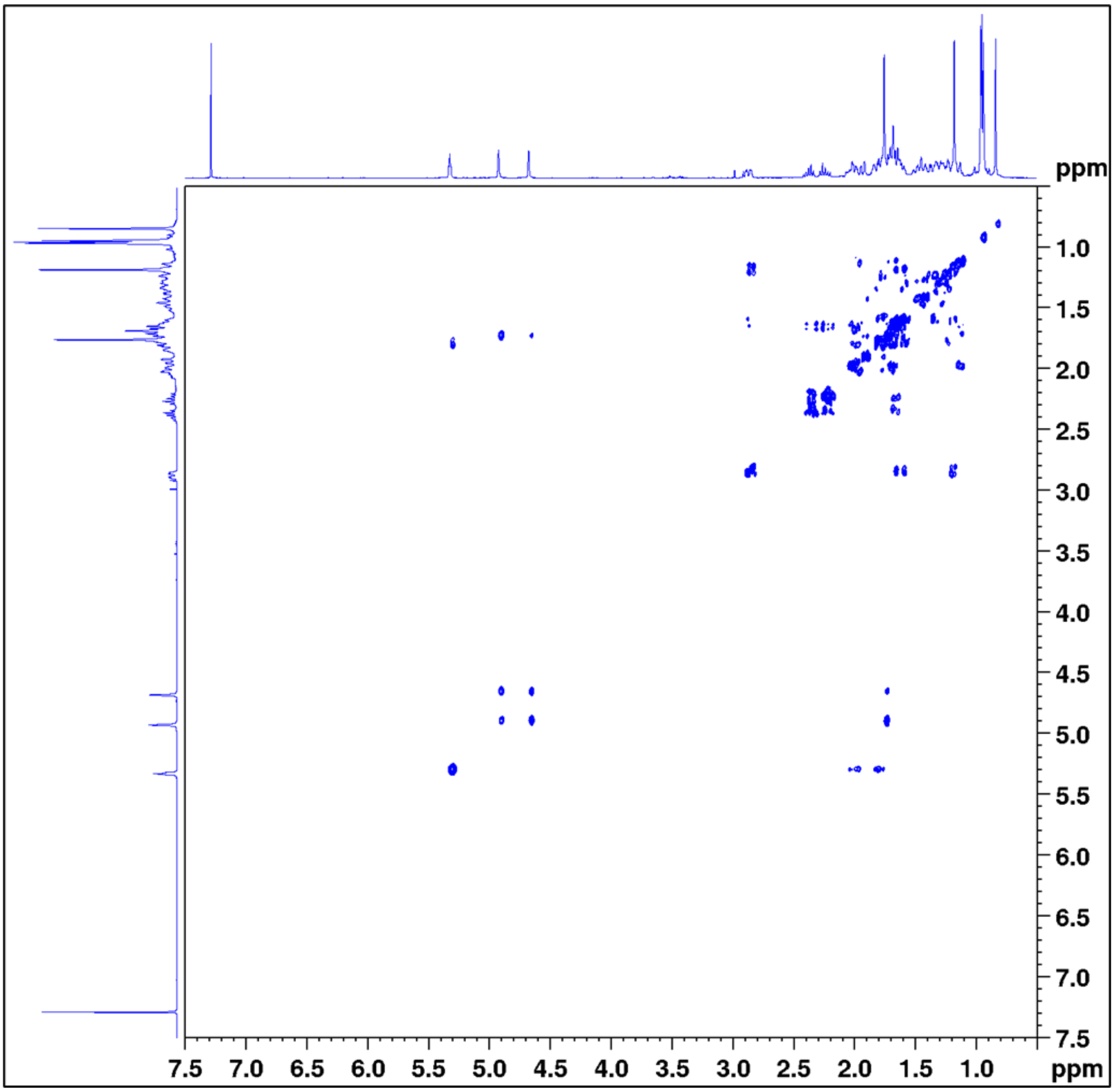

Figure S*15*: COSY (400 MHz, $CDCl_3$) spectrum of **8**.

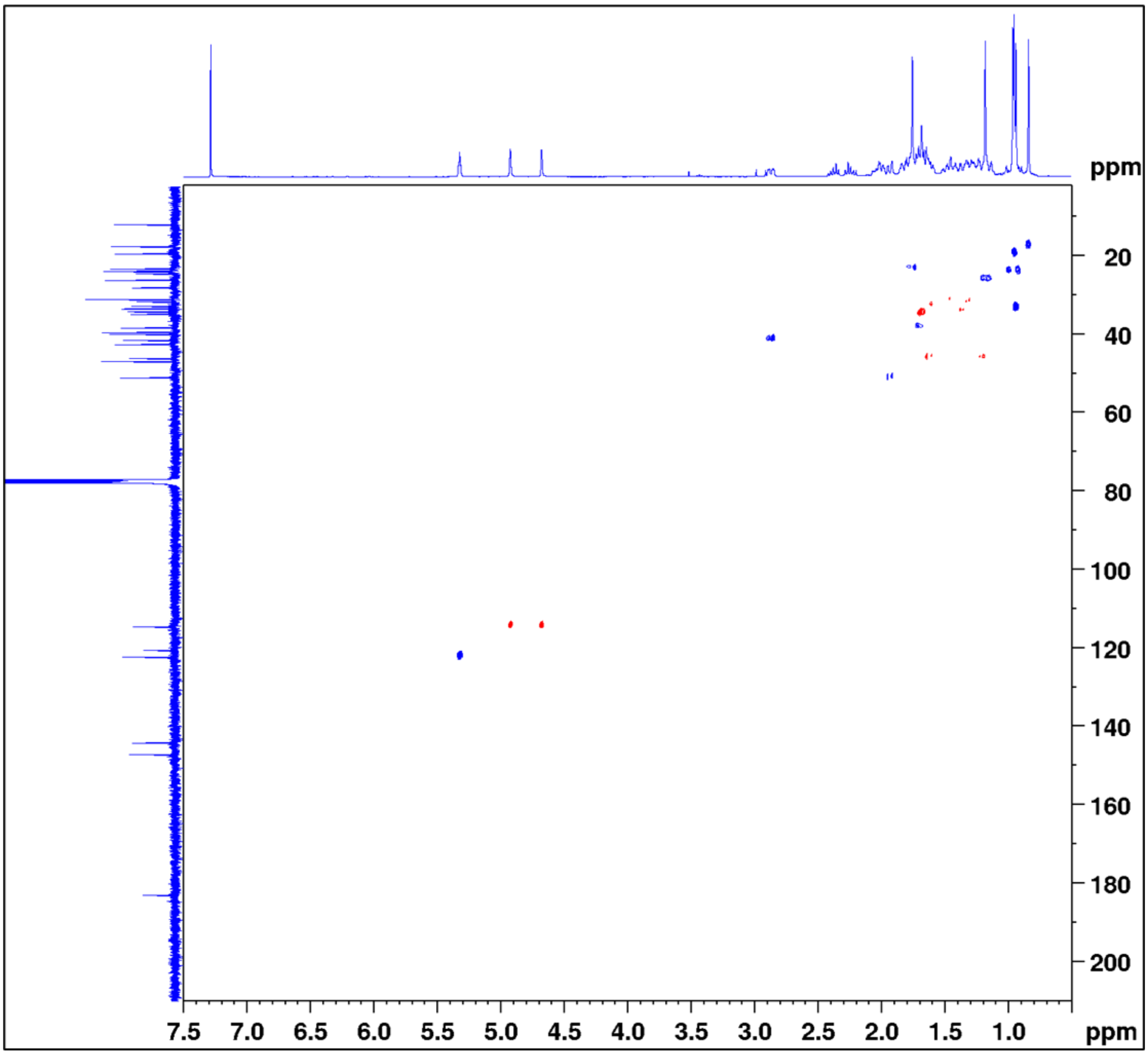

Figure S*16*: HSQC (400 MHz, $CDCl_3$) spectrum of **8**.

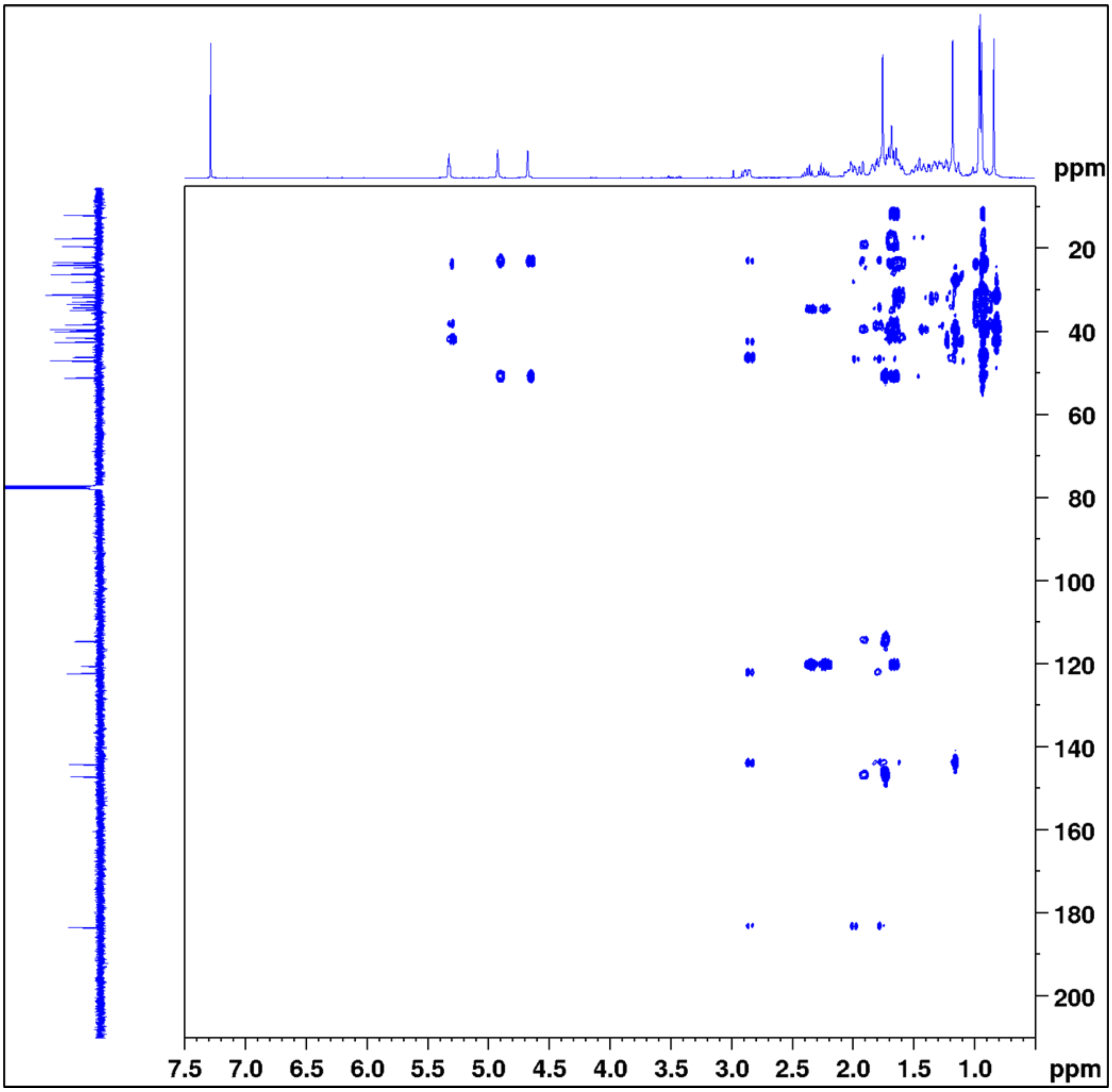

Figure S*17*: HMBC (400 MHz, $CDCl_3$) spectrum of **8**.

3-Oxo-3α-omo-3α-aza-olean-12-en-28-oic acid (**9**)

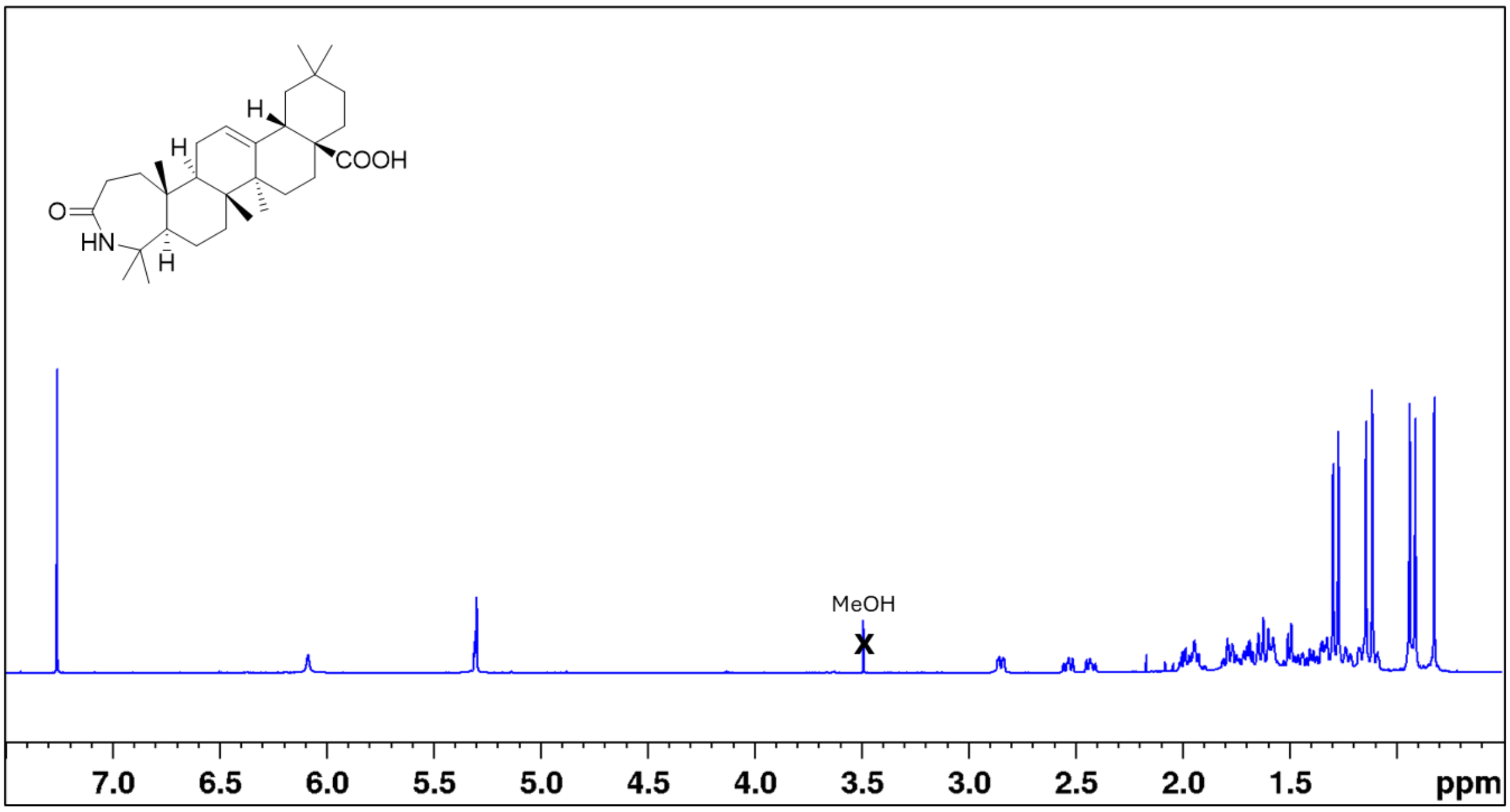

Figure S*18*: ^{1}H (600 MHz, $CDCl_3$) spectrum of **9**.

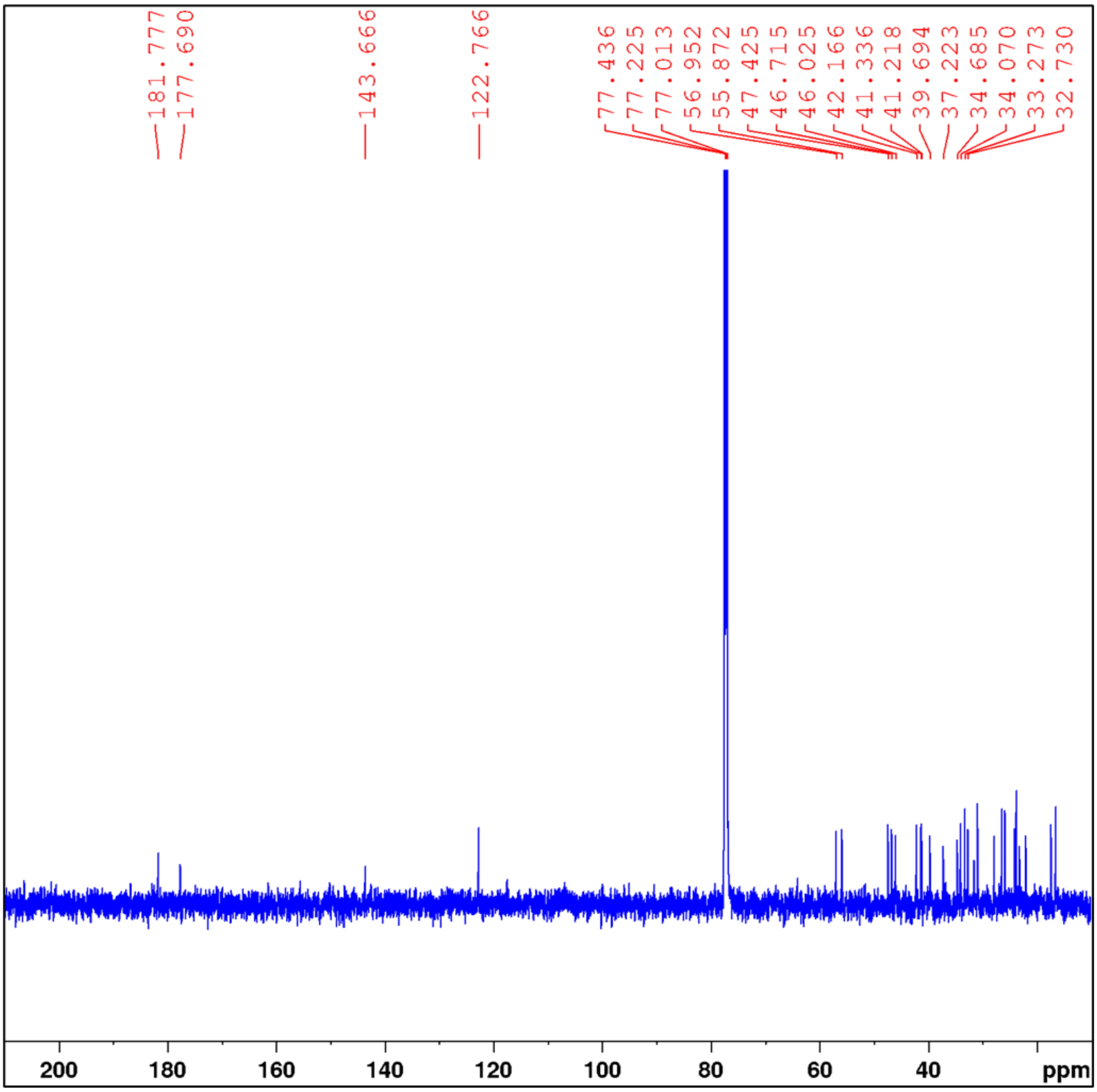

Figure S*19*: ^{13}C (600 MHz, $CDCl_3$) spectrum of **9**.

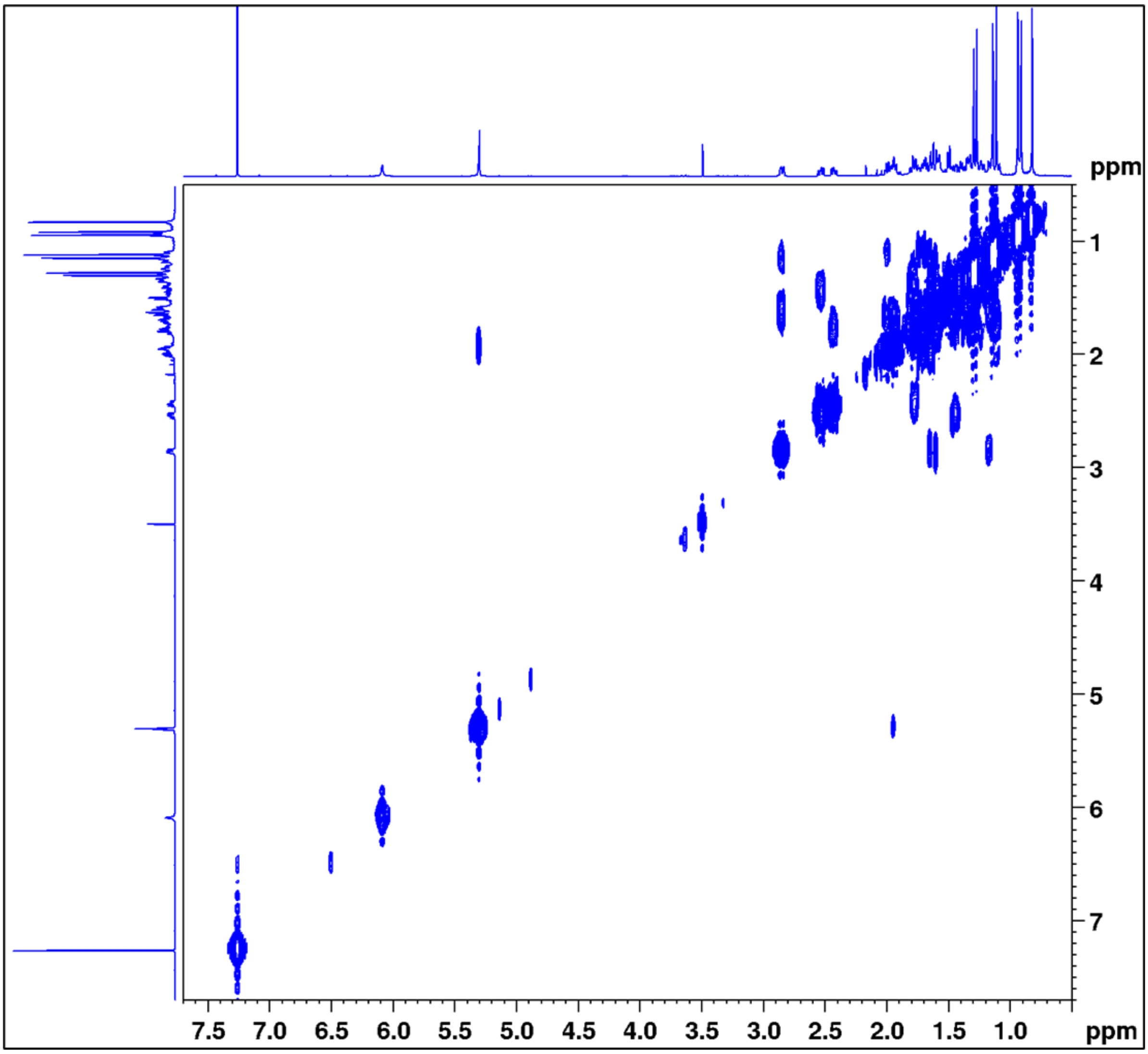

Figure S*20*: COSY (600 MHz, $CDCl_3$) spectrum of **9**.

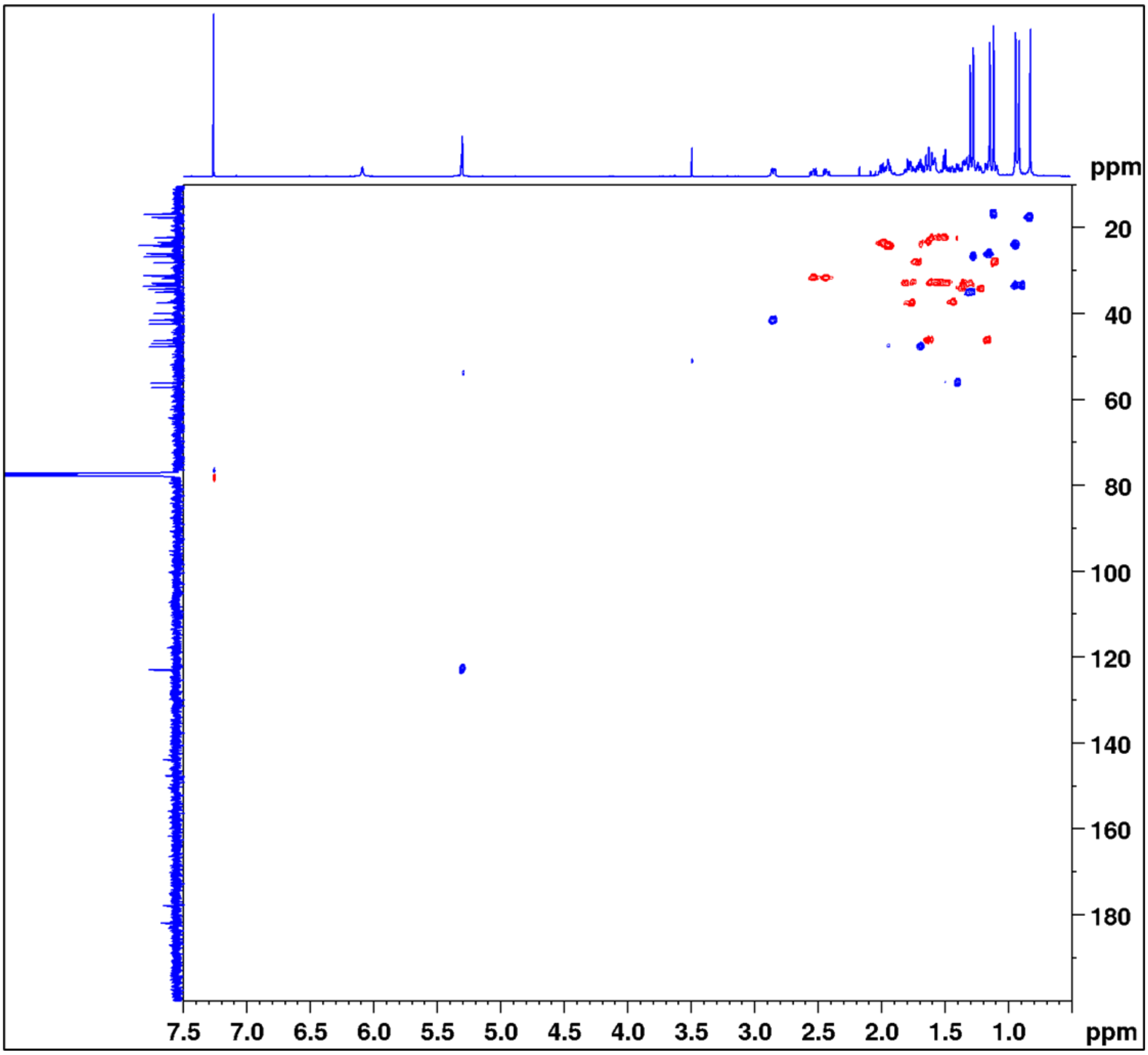

Figure S*21*: HSQC (600 MHz, $CDCl_3$) spectrum of **9**.

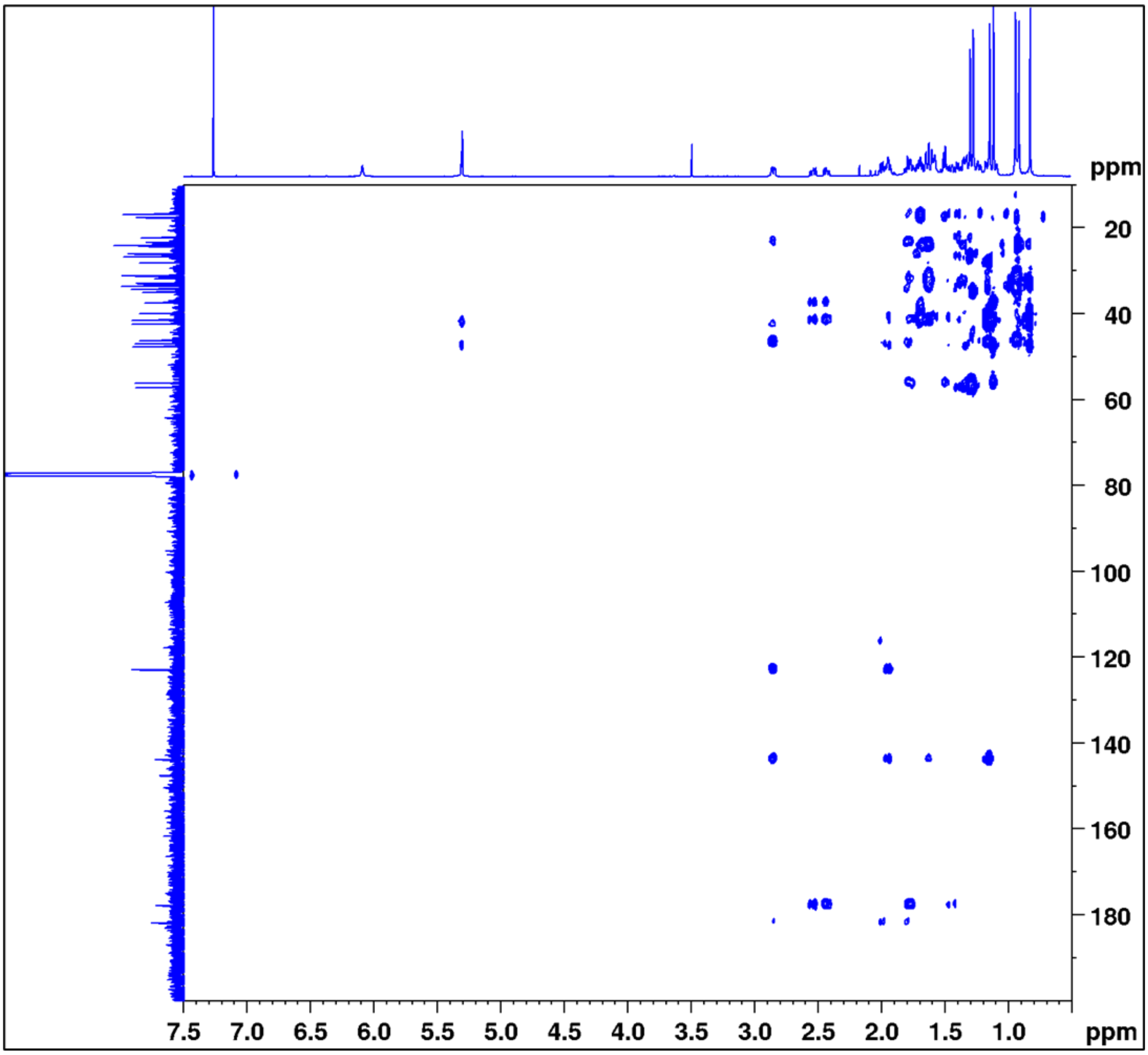

Figure S*22*: HMBC (600 MHz, $CDCl_3$) spectrum of **9**.
